# Supplementary material for: Critical node identification and resilience analysis against cascading failures
Source: PLoS One. 2026 Feb 27;21(2):e0344005. doi: 10.1371/journal.pone.0344005 (PMC12948132; doi:10.1371/journal.pone.0344005)
Supplement: S1 File — (PDF) [file pone.0344005.s001.pdf]

Supplementary Material

Table S1 Topological centrality metrics and entropy values for all nodes in the network

Node	Degree _Centrality	Closeness_ Centrality	Eigenvector _Centrality	Betweenness _Centrality	Entropy	Node	Degree _Centrality	Closeness _Centrality	Eigenvector _Centrality	Betweenness _Centrality	Entropy
**1**	0.00906	0.31345	0.00311	0.00000	1.04339	**168**	0.02115	0.41118	0.02007	0.00000	1.73729
**2**	0.00906	0.31345	0.00260	0.00000	1.05303	**169**	0.04834	0.43381	0.03578	0.00082	2.56598
**3**	0.00604	0.31315	0.00237	0.00000	0.55327	**170**	0.01813	0.36057	0.00578	0.00000	1.72830
**4**	0.01511	0.35861	0.01203	0.00813	1.41733	**171**	0.00906	0.28315	0.00001	0.00304	1.07336
**5**	0.00604	0.31315	0.00238	0.00000	0.55178	**172**	0.10574	0.47218	0.08950	0.00834	3.44034
**6**	0.00906	0.31675	0.00192	0.00000	0.86200	**173**	0.01208	0.40415	0.01289	0.00000	1.22100
**7**	0.00906	0.31675	0.00138	0.00000	0.95357	**174**	0.16012	0.47017	0.11761	0.00957	3.68488
**8**	0.08761	0.45467	0.11797	0.15330	3.09251	**175**	0.00302	0.34443	0.00079	0.00000	0.00000
**9**	0.01208	0.24320	0.00000	0.00000	1.33563	**176**	0.18429	0.49329	0.12521	0.01082	3.89482
**10**	0.01208	0.24320	0.00000	0.00302	1.28305	**177**	0.14502	0.48180	0.11796	0.01193	3.52370
**11**	0.00906	0.24302	0.00000	0.00000	1.01140	**178**	0.00302	0.34805	0.00087	0.00000	0.00000
**12**	0.00906	0.24302	0.00000	0.00000	0.97269	**179**	0.12689	0.44790	0.06828	0.00079	3.57074
**13**	0.04230	0.32012	0.00170	0.06529	2.02950	**180**	0.00302	0.34623	0.00036	0.00000	0.00000
**14**	0.00302	0.24267	0.00000	0.00000	0.00000	**181**	0.02417	0.41323	0.01676	0.00604	1.87873
**15**	0.00302	0.24267	0.00000	0.00000	0.00000	**182**	0.28399	0.52875	0.16000	0.11450	4.27520
**16**	0.00604	0.31345	0.00067	0.00082	0.68455	**183**	0.06949	0.44730	0.05756	0.01134	2.83582
**17**	0.00906	0.24302	0.00000	0.00000	1.08142	**184**	0.01208	0.37699	0.01277	0.00000	1.22242
**18**	0.00302	0.24267	0.00000	0.00000	0.00000	**185**	0.00302	0.34805	0.00045	0.00000	0.00000
**19**	0.00906	0.24302	0.00000	0.00000	0.89229	**186**	0.01208	0.39311	0.00402	0.00000	1.30254
**20**	0.00906	0.24302	0.00000	0.00000	0.88750	**187**	0.00302	0.33984	0.00077	0.00000	0.00000
**21**	0.00302	0.24267	0.00000	0.00000	0.00000	**188**	0.00302	0.29266	0.00002	0.00000	0.00000
**22**	0.00604	0.26544	0.00006	0.00522	0.69186	**189**	0.05438	0.43043	0.02099	0.01238	2.74455
**23**	0.00604	0.31315	0.00134	0.00000	0.46063	**190**	0.00906	0.34159	0.00129	0.00000	0.90635
**24**	0.00604	0.31315	0.00116	0.00000	0.49276	**191**	0.01511	0.39172	0.00451	0.00000	1.52983

**25**	0.00302	0.26501	0.00001	0.00000	0.00000	**192**	0.03021	0.40268	0.00693	0.00722	2.17968
**26**	0.02417	0.36017	0.00882	0.01860	1.73644	**193**	0.00302	0.34805	0.00032	0.00000	0.00000
**27**	0.00302	0.31285	0.00098	0.00000	0.00000	**194**	0.00302	0.30935	0.00029	0.00000	0.00000
**28**	0.00604	0.31315	0.00309	0.00000	0.59188	**195**	0.00604	0.34660	0.00045	0.00000	0.63386
**29**	0.01208	0.34769	0.00583	0.00000	0.93819	**196**	0.00302	0.34623	0.00050	0.00000	0.00000
**30**	0.00604	0.31315	0.00167	0.00000	0.54630	**197**	0.06042	0.43783	0.04508	0.00042	2.72928
**31**	0.00906	0.26565	0.00005	0.00064	0.97584	**198**	0.03625	0.41952	0.02054	0.00189	2.34102
**32**	0.00604	0.25859	0.00001	0.00540	0.58879	**199**	0.00604	0.34879	0.00041	0.00000	0.67737
**33**	0.01208	0.34769	0.00466	0.01077	1.12512	**200**	0.00302	0.28339	0.00003	0.00000	0.00000
**34**	0.00604	0.31315	0.00217	0.00000	0.48921	**201**	0.20544	0.53301	0.21549	0.07302	3.88203
**35**	0.00906	0.31345	0.00246	0.00005	0.96461	**202**	0.05740	0.42382	0.02446	0.00153	2.74424
**36**	0.00906	0.31345	0.00312	0.00007	0.93276	**203**	0.07855	0.44973	0.08635	0.00503	2.98285
**37**	0.00604	0.31315	0.00563	0.00000	0.49051	**204**	0.02115	0.39125	0.00502	0.00148	1.79792
**38**	0.00906	0.31345	0.00463	0.00005	0.93551	**205**	0.00604	0.34879	0.00053	0.00000	0.66991
**39**	0.00906	0.35101	0.00187	0.00000	0.85204	**206**	0.01813	0.37486	0.00557	0.00375	1.59530
**40**	0.02115	0.34018	0.00237	0.00000	1.76230	**207**	0.00604	0.34660	0.00057	0.00000	0.59059
**41**	0.00906	0.36334	0.00586	0.00000	0.92027	**208**	0.00302	0.33434	0.00166	0.00000	0.00000
**42**	0.00302	0.34515	0.00045	0.00000	0.00000	**209**	0.00604	0.34660	0.00100	0.00000	0.47414
**43**	0.00302	0.35101	0.00172	0.00000	0.00000	**210**	0.00302	0.33984	0.00123	0.00000	0.00000
**44**	0.00302	0.35101	0.00168	0.00000	0.00000	**211**	0.00302	0.34623	0.00069	0.00000	0.00000
**45**	0.05740	0.41952	0.02783	0.01529	2.65662	**212**	0.05740	0.42820	0.03103	0.00000	2.80792
**46**	0.02417	0.36374	0.00671	0.00197	1.77647	**213**	0.04532	0.39499	0.01563	0.01447	2.40503
**47**	0.17221	0.52623	0.21519	0.11266	3.80530	**214**	0.00604	0.35175	0.00212	0.00000	0.56300
**48**	0.00906	0.34805	0.00104	0.00000	0.94240	**215**	0.00906	0.36374	0.00297	0.00000	1.02027
**49**	0.01208	0.34660	0.00079	0.00287	1.30843	**216**	0.04834	0.42655	0.02251	0.00415	2.62135
**50**	0.03021	0.37025	0.01045	0.00135	1.96256	**217**	0.16918	0.47218	0.10759	0.02135	3.86226
**51**	0.01208	0.38895	0.00778	0.00000	1.28870	**218**	0.05438	0.42327	0.02219	0.00285	2.70967
**52**	0.00302	0.35101	0.00092	0.00000	0.00000	**219**	0.14804	0.49256	0.16852	0.00972	3.70699
**53**	0.01511	0.36696	0.00730	0.00319	1.47997	**220**	0.00906	0.36860	0.00272	0.00000	0.93107
**54**	0.02115	0.35326	0.00233	0.00441	1.73748	**221**	0.15106	0.44730	0.05993	0.01340	3.72900

**55**	0.02417	0.34053	0.00191	0.00173	1.83394	**222**	0.03021	0.42058	0.01293	0.00027	2.17074
**56**	0.01813	0.34916	0.00098	0.00075	1.75333	**223**	0.00604	0.35363	0.00108	0.00000	0.61086
**57**	0.01208	0.37959	0.00217	0.00084	1.31534	**224**	0.01208	0.37872	0.00700	0.00000	1.04817
**58**	0.02417	0.35363	0.00240	0.01081	1.90797	**225**	0.04230	0.42545	0.02271	0.00103	2.52262
**59**	0.03021	0.36214	0.00357	0.00946	1.99306	**226**	0.00906	0.36860	0.00268	0.00000	0.91767
**60**	0.02417	0.34989	0.00178	0.00722	1.91277	**227**	0.00302	0.33776	0.00055	0.00000	0.00000
**61**	0.01813	0.29713	0.00030	0.00000	1.64922	**228**	0.00604	0.34842	0.00125	0.00604	0.36520
**62**	0.03021	0.37025	0.00813	0.00359	2.16517	**229**	0.02115	0.40121	0.00748	0.00132	1.77112
**63**	0.02719	0.36983	0.00873	0.00809	1.88765	**230**	0.26284	0.50923	0.13589	0.05117	4.23377
**64**	0.00906	0.32611	0.00046	0.00000	0.89490	**231**	0.00302	0.33434	0.00065	0.00000	0.00000
**65**	0.12387	0.48110	0.09926	0.02619	3.40703	**232**	0.13293	0.47421	0.09759	0.02000	3.63319
**66**	0.01208	0.32611	0.00019	0.00000	1.21165	**233**	0.06949	0.44790	0.05073	0.00887	3.01866
**67**	0.23565	0.53997	0.19942	0.08161	4.16852	**234**	0.00604	0.35363	0.00127	0.00000	0.66895
**68**	0.01813	0.38399	0.00807	0.00006	1.57188	**235**	0.00604	0.33810	0.00041	0.00000	0.69217
**69**	0.01208	0.37959	0.00141	0.00000	1.24434	**236**	0.00604	0.34842	0.00142	0.00000	0.33830
**70**	0.00906	0.38002	0.00244	0.00123	1.08664	**237**	0.01511	0.35745	0.00458	0.00000	1.33546
**71**	0.02719	0.38668	0.00373	0.01039	2.02031	**238**	0.00302	0.33776	0.00056	0.00000	0.00000
**72**	0.02115	0.39125	0.01077	0.00000	1.86644	**239**	0.03021	0.38895	0.00933	0.00343	2.22279
**73**	0.01813	0.38046	0.00143	0.00220	1.56713	**240**	0.02417	0.41118	0.01005	0.00057	1.85834
**74**	0.01511	0.35900	0.00520	0.00000	1.40332	**241**	0.00302	0.35707	0.00078	0.00000	0.00000
**75**	0.02719	0.37443	0.00933	0.01152	1.88449	**242**	0.02115	0.38713	0.01120	0.00347	1.43626
**76**	0.01813	0.39125	0.00840	0.00335	1.70632	**243**	0.00906	0.34301	0.00256	0.00000	0.92653
**77**	0.01208	0.37959	0.00122	0.00032	1.15609	**244**	0.00302	0.34443	0.00025	0.00000	0.00000
**78**	0.00906	0.38355	0.00193	0.00000	0.88553	**245**	0.05136	0.40713	0.02650	0.00459	2.46974
**79**	0.00906	0.38355	0.00188	0.00306	0.96436	**246**	0.06949	0.45972	0.06995	0.00630	2.80222
**80**	0.03021	0.39358	0.01456	0.00279	2.12595	**247**	0.00302	0.35707	0.00057	0.00000	0.00000
**81**	0.01511	0.40613	0.01403	0.00635	1.30925	**248**	0.17825	0.52456	0.21842	0.03558	3.81636
**82**	0.03323	0.40073	0.01044	0.00706	2.29075	**249**	0.02719	0.39736	0.01046	0.00058	1.87888
**83**	0.04532	0.41635	0.02267	0.03166	2.46964	**250**	0.04230	0.42545	0.03012	0.00340	2.31118
**84**	0.00604	0.37959	0.00170	0.00000	0.64645	**251**	0.02115	0.38759	0.01311	0.00000	1.76202

**85**	0.01511	0.37872	0.00659	0.00553	1.02033	**252**	0.01208	0.35326	0.00501	0.00000	1.23594
**86**	0.00906	0.35668	0.00371	0.00000	0.90875	**253**	0.06647	0.45342	0.06790	0.00003	2.77199
**87**	0.00906	0.32579	0.00054	0.00000	1.02953	**254**	0.01813	0.35939	0.00209	0.00007	1.75739
**88**	0.00302	0.37829	0.00070	0.00000	0.00000	**255**	0.30514	0.53560	0.17557	0.05925	4.39021
**89**	0.00302	0.32012	0.00107	0.00000	0.00000	**256**	0.05136	0.42545	0.01907	0.00787	2.69077
**90**	0.02719	0.40219	0.01009	0.00626	1.93783	**257**	0.00302	0.35707	0.00073	0.00000	0.00000
**91**	0.05438	0.40663	0.02241	0.00000	2.74191	**258**	0.18127	0.50152	0.16844	0.03455	3.91431
**92**	0.06042	0.41272	0.03021	0.00000	2.78413	**259**	0.01208	0.35477	0.00123	0.00033	1.33395
**93**	0.01208	0.38533	0.00241	0.00000	1.24365	**260**	0.03323	0.39832	0.01444	0.00006	2.17768
**94**	0.10272	0.46685	0.10572	0.00696	3.28105	**261**	0.35650	0.55444	0.18936	0.14443	4.59608
**95**	0.06647	0.41323	0.03137	0.00707	2.90524	**262**	0.03927	0.33914	0.00453	0.00030	2.53250
**96**	0.02115	0.39125	0.00931	0.00071	1.81497	**263**	0.10876	0.48180	0.13376	0.00526	3.35023
**97**	0.00906	0.30311	0.00012	0.00093	0.99861	**264**	0.00302	0.33434	0.00060	0.00000	0.00000
**98**	0.00604	0.33741	0.00104	0.00113	0.65908	**265**	0.00302	0.34916	0.00046	0.00000	0.00000
**99**	0.02719	0.39641	0.00907	0.00212	2.07307	**266**	0.02115	0.37025	0.00300	0.00146	1.84477
**100**	0.02115	0.38668	0.00257	0.00139	1.76991	**267**	0.02115	0.37025	0.00311	0.00000	1.83039
**101**	0.04230	0.40317	0.01976	0.00322	2.48706	**268**	0.00302	0.35707	0.00064	0.00000	0.00000
**102**	0.01208	0.38533	0.00261	0.00331	1.18052	**269**	0.00604	0.35745	0.00057	0.00000	0.52186
**103**	0.00604	0.34018	0.00089	0.00000	0.37282	**270**	0.00604	0.35745	0.00044	0.00000	0.57838
**104**	0.00906	0.33879	0.00067	0.00963	1.05932	**271**	0.02719	0.37233	0.00620	0.00223	2.04575
**105**	0.00906	0.34879	0.00296	0.00000	1.04975	**272**	0.01511	0.35477	0.00206	0.00000	1.53084
**106**	0.01813	0.39311	0.00603	0.00033	1.66265	**273**	0.05136	0.42058	0.03052	0.00138	2.60230
**107**	0.00604	0.37872	0.00074	0.00082	0.65823	**274**	0.03927	0.42005	0.03618	0.00000	2.40050
**108**	0.01208	0.35401	0.00308	0.00000	1.19450	**275**	0.01813	0.35939	0.00266	0.00121	1.76829
**109**	0.15106	0.48392	0.17311	0.00069	3.66732	**276**	0.05136	0.42710	0.03470	0.00503	2.72210
**110**	0.00604	0.34479	0.00242	0.00000	0.66618	**277**	0.00302	0.35707	0.00055	0.00000	0.00000
**111**	0.02115	0.39079	0.00453	0.00484	1.78647	**278**	0.00302	0.35707	0.00125	0.00000	0.00000
**112**	0.21148	0.52373	0.16054	0.02005	3.93228	**279**	0.00302	0.35707	0.00103	0.00000	0.00000
**113**	0.00604	0.34194	0.00171	0.00000	0.69245	**280**	0.00302	0.35707	0.00079	0.00000	0.00000
**114**	0.00302	0.37829	0.00032	0.00000	0.00000	**281**	0.03021	0.37699	0.00826	0.00255	2.16830

**115**	0.00604	0.34018	0.00077	0.00153	0.40537	**282**	0.00302	0.35707	0.00100	0.00000	0.00000
**116**	0.01813	0.32675	0.00086	0.00007	1.69672	**283**	0.02719	0.37233	0.00693	0.00070	2.10766
**117**	0.00302	0.33984	0.00050	0.00000	0.00000	**284**	0.06647	0.42987	0.04121	0.00284	2.93374
**118**	0.41994	0.60734	0.20708	0.11227	4.66656	**285**	0.00302	0.32198	0.00109	0.00000	0.00000
**119**	0.08761	0.44016	0.06995	0.00020	3.09934	**286**	0.03021	0.37742	0.01117	0.00161	2.21181
**120**	0.02417	0.39736	0.01164	0.01074	1.68192	**287**	0.01511	0.35630	0.00650	0.00000	1.56187
**121**	0.00302	0.28584	0.00037	0.00000	0.00000	**288**	0.05438	0.42655	0.03494	0.00201	2.64069
**122**	0.00302	0.27885	0.00001	0.00000	0.00000	**289**	0.00302	0.34916	0.00139	0.00000	0.00000
**123**	0.07855	0.44133	0.05959	0.00471	3.06547	**290**	0.00604	0.35784	0.00225	0.00000	0.62799
**124**	0.00604	0.34842	0.00208	0.00000	0.43470	**291**	0.00302	0.35707	0.00147	0.00000	0.00000
**125**	0.04532	0.40514	0.02656	0.00004	2.56504	**292**	0.09970	0.45845	0.10013	0.00562	3.38559
**126**	0.02417	0.39452	0.00580	0.00461	1.92250	**293**	0.18731	0.49403	0.15445	0.01964	3.98090
**127**	0.01208	0.39452	0.00490	0.00000	1.09355	**294**	0.00302	0.35707	0.00143	0.00000	0.00000
**128**	0.02719	0.41899	0.01823	0.00080	2.01179	**295**	0.00604	0.35363	0.00335	0.00000	0.68200
**129**	0.01511	0.39641	0.00923	0.00000	1.45077	**296**	0.08157	0.43268	0.05416	0.00555	3.10644
**130**	0.02719	0.39593	0.01017	0.00619	1.69906	**297**	0.05740	0.43553	0.04424	0.00234	2.75959
**131**	0.13595	0.47626	0.12026	0.00475	3.57597	**298**	0.02115	0.35939	0.00938	0.00028	1.61688
**132**	0.01208	0.36454	0.00702	0.00000	1.22713	**299**	0.16918	0.47695	0.15578	0.00499	3.86511
**133**	0.04532	0.43211	0.03445	0.00478	2.52694	**300**	0.02417	0.35978	0.01108	0.00000	1.76766
**134**	0.00604	0.37872	0.00378	0.00000	0.21726	**301**	0.12689	0.45342	0.12462	0.00891	3.56371
**135**	0.00302	0.28242	0.00001	0.00000	0.00000	**302**	0.00906	0.32643	0.00497	0.00000	0.91393
**136**	0.01511	0.38987	0.01158	0.00533	1.36654	**303**	0.00906	0.35823	0.00334	0.00000	1.02327
**137**	0.03021	0.39784	0.00589	0.00217	2.07118	**304**	0.00302	0.35707	0.00236	0.00000	0.00000
**138**	0.00906	0.34879	0.00205	0.00537	0.96174	**305**	0.05740	0.42820	0.05732	0.00000	2.76310
**139**	0.02417	0.39311	0.00606	0.00686	1.94485	**306**	0.08157	0.43325	0.08097	0.00075	3.12588
**140**	0.01813	0.39311	0.00559	0.00539	1.64270	**307**	0.08761	0.44016	0.08847	0.00057	3.16385
**141**	0.00604	0.32675	0.00053	0.00000	0.62072	**308**	0.01511	0.35900	0.00534	0.00000	1.57493
**142**	0.05136	0.39976	0.02472	0.01058	2.76723	**309**	0.00604	0.35784	0.00433	0.00000	0.66338
**143**	0.03927	0.40073	0.02651	0.00091	2.25381	**310**	0.10272	0.44370	0.10972	0.00383	3.37437
**144**	0.17825	0.50767	0.12228	0.06111	3.91070	**311**	0.14199	0.47695	0.17357	0.01813	3.69371

**145**	0.01208	0.38533	0.00175	0.00000	1.27337	**312**	0.00906	0.32356	0.00075	0.00000	1.01852
**146**	0.15106	0.45467	0.09018	0.00146	3.70111	**313**	0.07251	0.47626	0.18494	0.05116	2.85059
**147**	0.20242	0.49329	0.15652	0.00779	3.92671	**314**	0.00906	0.32356	0.00024	0.00000	1.09439
**148**	0.01813	0.39358	0.00466	0.00423	1.35288	**315**	0.00906	0.32356	0.00033	0.00150	0.84790
**149**	0.02115	0.39976	0.01159	0.00000	1.80544	**316**	0.03021	0.36737	0.04003	0.01201	1.39093
**150**	0.13897	0.48041	0.14778	0.00075	3.52725	**317**	0.01208	0.32387	0.00034	0.00000	1.27942
**151**	0.01511	0.35439	0.00263	0.00395	1.54843	**318**	0.01813	0.35745	0.02538	0.00491	1.11047
**152**	0.28399	0.51398	0.13934	0.08780	4.26655	**319**	0.00906	0.32356	0.00108	0.00000	0.96491
**153**	0.02719	0.41272	0.03355	0.00000	2.09487	**320**	0.01208	0.33468	0.01946	0.00000	1.08911
**154**	0.01208	0.38578	0.00143	0.00155	1.32413	**321**	0.06344	0.42655	0.11025	0.01398	2.84324
**155**	0.00604	0.37915	0.00077	0.00101	0.66131	**322**	0.02719	0.35784	0.03597	0.00000	1.95997
**156**	0.00302	0.33984	0.00091	0.00000	0.00000	**323**	0.00302	0.29928	0.00016	0.00000	0.00000
**157**	0.02719	0.40073	0.01067	0.00511	1.96562	**324**	0.01511	0.33536	0.02016	0.00064	1.13340
**158**	0.01813	0.39218	0.00310	0.00921	1.63678	**325**	0.02417	0.34018	0.02969	0.00000	1.82059
**159**	0.08459	0.44430	0.06817	0.00872	3.06843	**326**	0.00302	0.32293	0.00322	0.00000	0.00000
**160**	0.00604	0.34660	0.00050	0.00000	0.68850	**327**	0.01208	0.24646	0.00006	0.00604	0.71378
**161**	0.08761	0.44251	0.04218	0.00810	3.08770	**328**	0.00604	0.24610	0.00002	0.00601	0.68316
**162**	0.18731	0.48180	0.13471	0.00607	3.86290	**329**	0.01208	0.32547	0.01318	0.02397	0.73629
**163**	0.01208	0.38623	0.00181	0.00617	1.35241	**330**	0.00604	0.24610	0.00025	0.00000	0.68928
**164**	0.00906	0.38533	0.00152	0.00000	0.99101	**331**	0.00604	0.34916	0.04269	0.00000	0.67066
**165**	0.00302	0.34805	0.00107	0.00000	0.00000	**332**	0.00302	0.19785	0.00000	0.00000	0.00000
**166**	0.25680	0.52456	0.17165	0.11505	4.31979						
**167**	0.12387	0.46165	0.09233	0.01055	3.47824						

Table S2. Unified training parameters for model comparison.

Parameter	Value
**Input Feature Dimension**	2<br>(P1,P2)
**Output Dimension**	1<br>(Node criticality score)
**Optimizer**	Adam
**Learning Rate**	0.005
**Maximum Epochs**	200
**Train/Val/Test Split**	60%/20%/20%

Table S3. Model-Specific hyperparameters

Parameter	Value		
	**GCN**	**GraphSAGE**	**GAT**
**Hidden Dimension**	64	64	32
**Heads/Layers**	2 layers	2 layers	8 heads (L1), 1 head (L2)
**Dropout Rate**	0.5	0.5	0.6
**Batch Normalization**	No	Yes	No
**Label-aware Attention**	No	No	Yes
**Loss Function**	PairwiseRanking	PairwiseRanking	Weighted PairwiseRankingLoss
**Early Stopping**	15	15	20
**Early Stopping Metric**	Validation Loss	Validation Loss	Spearman Correlation
**Weight Decay**	1×10^{-4}	1×10^{-4}	5×10^{-4}

Table S4. Evaluation metrics comparison of three GNN models

Classification	Performance Metrics	GNC	GraphSAGE	GAT	Optimal Range	Direction	Interpretation
**Effect Size**	**Cohen's d**	4.703	**2.814**	2.561	≥0.5 (medium), ≥0.8 (large)	Larger is better	Standardized difference between critical and ordinary nodes.
**Ranking Consistency**	**Spearman's ρ (95% CI)**	[0.738, 0.825]	**[0.777, 0.855]**	[0.643, 0.757]	≥0.7 with narrow CI (<0.2 width)	Larger is better	Strong rank correlation with stable estimation.
**Ranking Quality**	**Spearman correlation**	0.784	**0.822**	0.703			
	**NDCG@K**	0.959	**0.918**	0.800	≥0.8	Larger is better	Excellent ranking quality considering position discount.
	**F1@K**	0.939	**0.879**	0.800	≥0.7	Larger is better	Balanced precision and recall at top-K.
**Top-K Identification Accuracy**	**Precision@K**	0.939	**0.879**	0.800	≥0.7	Larger is better	High accuracy in top-K predictions.
	**Recall@K**	0.939	**0.879**	0.800	≥0.6	Larger is better	Adequate coverage of true critical nodes.
	**Accuracy**	0.403	**0.791**	0.612	Accuracy values should be interpreted in conjunction with precision and recall.	Larger is better	The proportion of correctly classified observations among all observations.
**Ranking Agreement**	**Overlap**	0.939	**0.879**	0.800	≥0.6	Larger is better	Substantial agreement between methods.
**Computational Efficiency**	**Inference time**	0.002s	**0.002s**	0.006s	<5s (for ~1000 nodes)	Smaller is better	Computational efficiency for practical deployment.

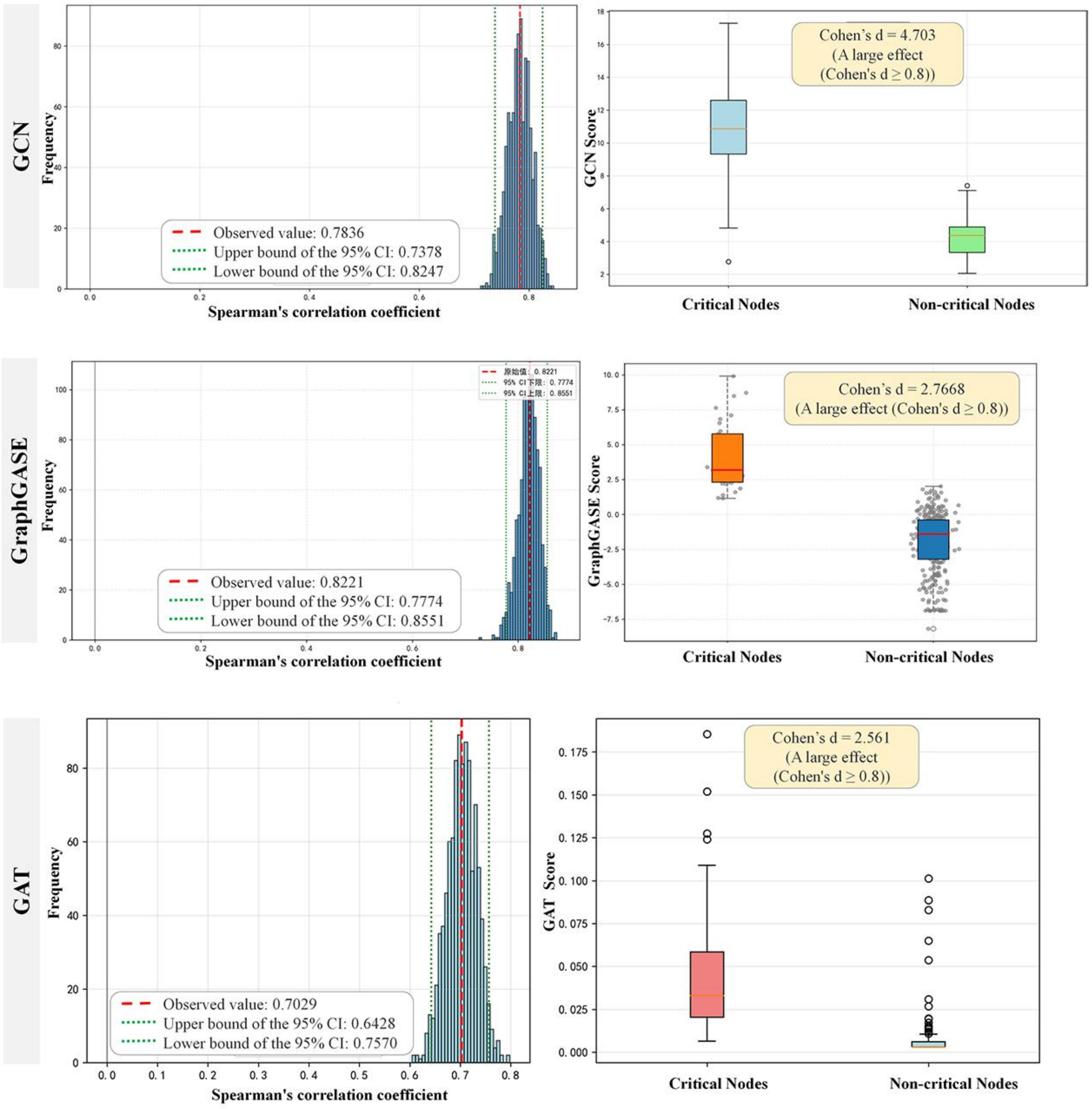

Figure S1. Bootstrapped Significance Spearman's Correlation Coefficient Distribution (1000 Resamples) of three GNN models

Table S5. Changes in evaluation metrics of the GraphSAGE model under β-ablation experiments.

Classification	Effect Size	Ranking Consistency		Ranking Quality				Top-K Identification Accuracy			Ranking Agreement	Computational Efficiency
**β**	**Cohen_d**	**SpearmanCI_Lower**	**Spearman CI_Upper**	**Spearman correlation**	**Spearman p_value**	**NDCG@33**	**F1@33**	**Precision@33**	**Recall@33**	**Accuracy@33**	**Overlap**	**Inference time**
**0.1**	0.974	0.372	0.542	0.461	7.040E-19	0.502	0.523	0.523	0.523	0.687	0.523	0.007s
**0.3**	0.239	0.198	0.394	0.299	2.688E-08	0.425	0.459	0.459	0.459	0.645	0.459	0.005s
**0.5**	0.533	0.207	0.402	0.308	1.020E-08	0.433	0.459	0.459	0.459	0.645	0.459	0.005s
**0.7**	0.532	0.194	0.391	0.296	3.997E-08	0.436	0.431	0.431	0.431	0.627	0.431	0.007s
**0.9**	0.529	0.189	0.387	0.291	6.568E-08	0.442	0.440	0.440	0.440	0.633	0.440	0.006s
**1.1**	0.514	0.184	0.382	0.286	1.159E-07	0.443	0.450	0.450	0.450	0.639	0.450	0.010s
**1.3**	0.514	0.183	0.381	0.285	1.265E-07	0.446	0.459	0.459	0.459	0.645	0.459	0.006s
**1.5**	0.511	0.181	0.380	0.283	1.490E-07	0.453	0.450	0.450	0.450	0.639	0.450	0.006s
**1.7**	0.511	0.180	0.378	0.282	1.772E-07	0.456	0.468	0.468	0.468	0.651	0.468	0.006s
**1.9**	0.495	0.173	0.372	0.276	3.366E-07	0.456	0.468	0.468	0.468	0.651	0.468	0.135s
**Averaging Across β Values(β∈[0.1, 2.0])**	0.821	0.780	0.854	2.928	0.000	0.959	0.939	0.939	0.939	0.821	0.939	0.002s

Table S6. LCC_Ratio——Targeted vs. Random Attacks: GraphSAGE versus Baseline Methods

Removed _Ratio	GraphSAGE _Targeted_Attack	GAT _Targeted_Attack	GCN _Targeted_Attack	S_value _Targeted_attack	Random _Attack
0.0030	0.9548	0.9970	0.9910	0.9548	0.9970
0.0060	0.9458	0.9880	0.9458	0.9458	0.9940
0.0090	0.9217	0.9789	0.9247	0.9217	0.9910
0.0120	0.8404	0.9759	0.9157	0.9006	0.9880
0.0151	0.8193	0.9548	0.8946	0.8916	0.9849
0.0181	0.8133	0.9518	0.8705	0.8705	0.9819
0.0211	0.8042	0.9337	0.8645	0.7892	0.9789
0.0241	0.7861	0.9127	0.8434	0.7831	0.9759
0.0271	0.7651	0.9066	0.8253	0.7651	0.9729
0.0301	0.7440	0.9036	0.8223	0.7440	0.9699
0.0331	0.7440	0.8976	0.8193	0.7048	0.9669
0.0361	0.7048	0.8916	0.8072	0.7018	0.9639
0.0392	0.7018	0.8886	0.8012	0.6958	0.9608
0.0422	0.6898	0.8283	0.7952	0.6958	0.9578
0.0452	0.6777	0.8253	0.7831	0.6837	0.9548
0.0482	0.6657	0.8223	0.7711	0.6717	0.9518
0.0512	0.6536	0.7982	0.7590	0.6596	0.9488
0.0542	0.6476	0.7831	0.7349	0.6476	0.9458
0.0572	0.6446	0.7711	0.7229	0.6446	0.9428
0.0602	0.6205	0.7590	0.6084	0.6325	0.9398
0.0633	0.6175	0.7530	0.6054	0.6265	0.9367
0.0663	0.6054	0.7500	0.5934	0.6145	0.9307
0.0693	0.5602	0.7410	0.5813	0.6024	0.9247
0.0723	0.5572	0.7380	0.5783	0.5994	0.9217
0.0753	0.5452	0.7349	0.5753	0.5753	0.9187
0.0783	0.5331	0.7319	0.5753	0.5301	0.9157
0.0813	0.5271	0.7229	0.5663	0.5271	0.9127
0.0843	0.5241	0.7199	0.5633	0.5181	0.9096
0.0873	0.5211	0.7139	0.5572	0.5151	0.9066
0.0904	0.5120	0.7108	0.5542	0.5060	0.9036
0.0934	0.5030	0.7078	0.5090	0.5030	0.9006
0.0964	0.5000	0.7018	0.5060	0.5000	0.8976
0.0994	0.4819	0.6988	0.4970	0.4940	0.8795
0.1024	0.4789	0.6837	0.4940	0.4880	0.8765
0.1054	0.4759	0.6807	0.4880	0.4759	0.8735
0.1084	0.4729	0.6777	0.4849	0.4729	0.8705
0.1114	0.4669	0.6747	0.4819	0.4699	0.8675
0.1145	0.4669	0.6717	0.4759	0.4639	0.8645

Removed _Ratio	GraphSAGE _Targeted_Attack	GAT _Targeted_Attack	GCN _Targeted_Attack	S_value _Targeted_attack	Random _Attack
0.1175	0.4608	0.6687	0.4096	0.4639	0.8614
0.1205	0.4578	0.6657	0.4036	0.4608	0.8584
0.1235	0.4096	0.6627	0.3946	0.4608	0.8554
0.1265	0.4006	0.6596	0.3946	0.4608	0.8524
0.1295	0.3886	0.6566	0.3434	0.4548	0.8494
0.1325	0.3855	0.6536	0.3434	0.4458	0.8464
0.1355	0.3795	0.6506	0.3404	0.4398	0.8434
0.1386	0.3795	0.6476	0.3373	0.4398	0.8404
0.1416	0.3102	0.6175	0.3253	0.4367	0.8373
0.1446	0.2590	0.6114	0.3072	0.4247	0.8343
0.1476	0.2470	0.6084	0.2169	0.4247	0.8313
0.1506	0.2440	0.5994	0.2169	0.4157	0.8283
0.1536	0.2259	0.5904	0.2169	0.4066	0.8253
0.1566	0.2259	0.5813	0.2169	0.4036	0.8223
0.1596	0.2169	0.5783	0.2169	0.4036	0.8193
0.1627	0.1325	0.5723	0.2139	0.3976	0.8163
0.1657	0.1325	0.5693	0.2018	0.3886	0.8102
0.1687	0.1325	0.5663	0.2018	0.3886	0.8072
0.1717	0.1325	0.5633	0.1928	0.3825	0.8012
0.1747	0.1325	0.5512	0.1928	0.3072	0.7982
0.1777	0.1325	0.4970	0.1325	0.2018	0.7952
0.1807	0.1325	0.4066	0.1325	0.2018	0.7922
0.1837	0.1265	0.4036	0.1325	0.2018	0.7892
0.1867	0.0723	0.4036	0.1325	0.2018	0.7861
0.1898	0.0723	0.3554	0.1325	0.2018	0.7831
0.1928	0.0723	0.3554	0.1325	0.2018	0.7801
0.1958	0.0693	0.3554	0.1325	0.2018	0.7771
0.1988	0.0482	0.3554	0.1325	0.2018	0.7741
0.2018	0.0482	0.3524	0.0753	0.2018	0.7711
0.2048	0.0482	0.3012	0.0753	0.2018	0.7681
0.2078	0.0482	0.3012	0.0753	0.2018	0.7651
0.2108	0.0482	0.2982	0.0753	0.1747	0.7620
0.2139	0.0482	0.2982	0.0663	0.1747	0.7590
0.2169	0.0482	0.2801	0.0663	0.1687	0.7560
0.2199	0.0452	0.2771	0.0482	0.1687	0.7530
0.2229	0.0392	0.2771	0.0482	0.1687	0.7500
0.2259	0.0392	0.2771	0.0482	0.1687	0.7470
0.2289	0.0392	0.2741	0.0482	0.1687	0.7440
0.2319	0.0392	0.2741	0.0482	0.1627	0.7410
0.2349	0.0392	0.2741	0.0392	0.1596	0.7380

Removed _Ratio	GraphSAGE _Targeted_Attack	GAT _Targeted_Attack	GCN _Targeted_Attack	S_value _Targeted_attack	Random _Attack
0.2380	0.0392	0.1596	0.0361	0.1596	0.7349
0.2410	0.0331	0.1596	0.0361	0.1596	0.7319
0.2440	0.0331	0.1596	0.0361	0.1596	0.7289
0.2470	0.0331	0.1596	0.0361	0.1596	0.7259
0.2500	0.0331	0.1596	0.0361	0.1386	0.7229
0.2530	0.0331	0.1596	0.0361	0.1386	0.7229
0.2560	0.0331	0.1596	0.0361	0.1325	0.7199
0.2590	0.0331	0.1596	0.0361	0.1295	0.7169
0.2620	0.0331	0.1596	0.0361	0.1205	0.7139
0.2651	0.0331	0.1596	0.0361	0.1205	0.7108
0.2681	0.0331	0.1596	0.0361	0.1205	0.7078
0.2711	0.0331	0.1596	0.0361	0.1205	0.7018
0.2741	0.0331	0.1596	0.0361	0.1205	0.6988
0.2771	0.0331	0.1596	0.0361	0.1175	0.6958
0.2801	0.0331	0.1596	0.0331	0.0994	0.6928
0.2831	0.0331	0.1596	0.0331	0.0994	0.6898
0.2861	0.0271	0.1596	0.0331	0.0873	0.6867
0.2892	0.0271	0.1596	0.0331	0.0873	0.6837
0.2922	0.0271	0.1596	0.0331	0.0873	0.6807
0.2952	0.0271	0.1596	0.0331	0.0873	0.6777
0.2982	0.0271	0.1596	0.0331	0.0873	0.6747
0.3012	0.0271	0.1596	0.0331	0.0873	0.6657
0.3042	0.0211	0.1596	0.0331	0.0873	0.6627
0.3072	0.0211	0.1596	0.0271	0.0873	0.6596
0.3102	0.0211	0.1596	0.0271	0.0873	0.6536
0.3133	0.0211	0.1596	0.0271	0.0873	0.6506
0.3163	0.0211	0.1596	0.0271	0.0873	0.6476
0.3193	0.0211	0.1596	0.0271	0.0873	0.6446
0.3223	0.0211	0.1596	0.0271	0.0843	0.6416
0.3253	0.0211	0.1596	0.0271	0.0843	0.6386
0.3283	0.0211	0.1596	0.0211	0.0843	0.6355
0.3313	0.0211	0.1596	0.0211	0.0843	0.6325
0.3343	0.0211	0.1596	0.0211	0.0843	0.6295
0.3373	0.0211	0.1596	0.0211	0.0843	0.6295
0.3404	0.0211	0.1596	0.0211	0.0843	0.6114
0.3434	0.0211	0.1596	0.0211	0.0843	0.6084
0.3464	0.0211	0.1596	0.0211	0.0783	0.6084
0.3494	0.0211	0.1596	0.0211	0.0753	0.6054
0.3524	0.0211	0.1596	0.0211	0.0693	0.6024
0.3554	0.0211	0.1596	0.0211	0.0693	0.5994

Removed _Ratio	GraphSAGE _Targeted_Attack	GAT _Targeted_Attack	GCN _Targeted_Attack	S_value _Targeted_attack	Random _Attack
0.3584	0.0211	0.1596	0.0211	0.0693	0.5964
0.3614	0.0211	0.1596	0.0211	0.0633	0.5934
0.3645	0.0151	0.1596	0.0211	0.0633	0.5904
0.3675	0.0151	0.1596	0.0211	0.0633	0.5873
0.3705	0.0151	0.1596	0.0211	0.0633	0.5873
0.3735	0.0151	0.1566	0.0211	0.0512	0.5843
0.3765	0.0151	0.1536	0.0211	0.0482	0.5813
0.3795	0.0151	0.1536	0.0211	0.0482	0.5783
0.3825	0.0151	0.1536	0.0211	0.0482	0.5753
0.3855	0.0151	0.1536	0.0211	0.0482	0.5723
0.3886	0.0151	0.1536	0.0211	0.0482	0.5693
0.3916	0.0151	0.1536	0.0211	0.0241	0.5663
0.3946	0.0151	0.1536	0.0211	0.0241	0.5633
0.3976	0.0151	0.1536	0.0211	0.0241	0.5602
0.4006	0.0151	0.1536	0.0211	0.0241	0.5572
0.4036	0.0151	0.1536	0.0211	0.0241	0.5452
0.4066	0.0151	0.1536	0.0211	0.0241	0.5331
0.4096	0.0151	0.1506	0.0211	0.0241	0.5271
0.4127	0.0151	0.1506	0.0151	0.0241	0.5241
0.4157	0.0151	0.1205	0.0151	0.0241	0.5241
0.4187	0.0151	0.1205	0.0151	0.0241	0.5241
0.4217	0.0120	0.1205	0.0151	0.0241	0.5211
0.4247	0.0090	0.1205	0.0151	0.0241	0.5181
0.4277	0.0090	0.1205	0.0151	0.0241	0.5181
0.4307	0.0090	0.1205	0.0151	0.0241	0.4880
0.4337	0.0090	0.1175	0.0151	0.0241	0.4849
0.4367	0.0090	0.1175	0.0151	0.0241	0.4819
0.4398	0.0090	0.1175	0.0151	0.0241	0.4819
0.4428	0.0090	0.1175	0.0151	0.0241	0.4789
0.4458	0.0090	0.1175	0.0151	0.0241	0.4759
0.4488	0.0090	0.1175	0.0151	0.0241	0.4729
0.4518	0.0090	0.1175	0.0151	0.0241	0.4729
0.4548	0.0090	0.1175	0.0151	0.0241	0.4699
0.4578	0.0090	0.1175	0.0151	0.0241	0.4699
0.4608	0.0090	0.1175	0.0151	0.0241	0.4669
0.4639	0.0090	0.1175	0.0151	0.0241	0.4639
0.4669	0.0060	0.1175	0.0151	0.0241	0.4608
0.4699	0.0060	0.1114	0.0151	0.0241	0.4578
0.4729	0.0060	0.1084	0.0151	0.0241	0.4548
0.4759	0.0060	0.1084	0.0151	0.0241	0.4518

Removed _Ratio	GraphSAGE _Targeted_Attack	GAT _Targeted_Attack	GCN _Targeted_Attack	S_value _Targeted_attack	Random _Attack
0.4789	0.0060	0.1084	0.0151	0.0241	0.4488
0.4819	0.0060	0.1084	0.0151	0.0241	0.4458
0.4849	0.0060	0.1084	0.0151	0.0241	0.4428
0.4880	0.0060	0.1084	0.0151	0.0241	0.4398
0.4910	0.0060	0.1084	0.0090	0.0241	0.4367
0.4940	0.0060	0.1084	0.0090	0.0241	0.4307
0.4970	0.0060	0.1084	0.0090	0.0241	0.4277
0.5000	0.0060	0.1084	0.0090	0.0241	0.4247
0.5030	0.0060	0.1054	0.0090	0.0241	0.4217
0.5060	0.0060	0.1024	0.0090	0.0241	0.4187
0.5090	0.0060	0.0994	0.0060	0.0241	0.4157
0.5120	0.0060	0.0964	0.0060	0.0241	0.4127
0.5151	0.0060	0.0964	0.0060	0.0241	0.4006
0.5181	0.0060	0.0964	0.0060	0.0241	0.3976
0.5211	0.0060	0.0964	0.0060	0.0241	0.3946
0.5241	0.0060	0.0964	0.0060	0.0241	0.3946
0.5271	0.0060	0.0964	0.0060	0.0241	0.3916
0.5301	0.0060	0.0934	0.0060	0.0241	0.3886
0.5331	0.0060	0.0904	0.0060	0.0241	0.3855
0.5361	0.0060	0.0873	0.0060	0.0241	0.3855
0.5392	0.0060	0.0873	0.0060	0.0241	0.3825
0.5422	0.0060	0.0873	0.0060	0.0241	0.3795
0.5452	0.0060	0.0873	0.0060	0.0241	0.3765
0.5482	0.0060	0.0873	0.0060	0.0241	0.3735
0.5512	0.0060	0.0873	0.0060	0.0241	0.3705
0.5542	0.0060	0.0873	0.0060	0.0241	0.3675
0.5572	0.0060	0.0843	0.0060	0.0241	0.3645
0.5602	0.0060	0.0813	0.0060	0.0241	0.3614
0.5633	0.0060	0.0813	0.0060	0.0241	0.3584
0.5663	0.0060	0.0783	0.0060	0.0241	0.3584
0.5693	0.0060	0.0783	0.0060	0.0241	0.3494
0.5723	0.0060	0.0783	0.0060	0.0241	0.3464
0.5753	0.0060	0.0783	0.0060	0.0241	0.3464
0.5783	0.0060	0.0783	0.0060	0.0241	0.3434
0.5813	0.0060	0.0753	0.0060	0.0241	0.3404
0.5843	0.0060	0.0723	0.0060	0.0241	0.3404
0.5873	0.0060	0.0723	0.0060	0.0241	0.3373
0.5904	0.0060	0.0723	0.0060	0.0241	0.3373
0.5934	0.0060	0.0693	0.0060	0.0241	0.3373
0.5964	0.0060	0.0693	0.0060	0.0241	0.3343

Removed _Ratio	GraphSAGE _Targeted_Attack	GAT _Targeted_Attack	GCN _Targeted_Attack	S_value _Targeted_attack	Random _Attack
0.5994	0.0060	0.0693	0.0060	0.0241	0.3313
0.6024	0.0060	0.0693	0.0060	0.0241	0.3283
0.6054	0.0060	0.0512	0.0060	0.0241	0.3283
0.6084	0.0060	0.0512	0.0060	0.0241	0.3253
0.6114	0.0060	0.0512	0.0060	0.0241	0.3223
0.6145	0.0060	0.0512	0.0060	0.0241	0.3193
0.6175	0.0060	0.0512	0.0060	0.0241	0.3163
0.6205	0.0060	0.0512	0.0060	0.0241	0.3133
0.6235	0.0060	0.0452	0.0060	0.0241	0.3102
0.6265	0.0060	0.0422	0.0060	0.0241	0.3072
0.6295	0.0060	0.0422	0.0060	0.0241	0.3042
0.6325	0.0060	0.0422	0.0060	0.0241	0.2801
0.6355	0.0060	0.0422	0.0060	0.0241	0.2771
0.6386	0.0060	0.0422	0.0060	0.0241	0.2741
0.6416	0.0060	0.0422	0.0060	0.0241	0.2711
0.6446	0.0060	0.0392	0.0060	0.0241	0.2681
0.6476	0.0060	0.0392	0.0060	0.0241	0.2681
0.6506	0.0060	0.0392	0.0060	0.0241	0.2681
0.6536	0.0060	0.0392	0.0060	0.0241	0.2651
0.6566	0.0060	0.0361	0.0060	0.0241	0.2620
0.6596	0.0060	0.0361	0.0060	0.0241	0.2620
0.6627	0.0060	0.0361	0.0060	0.0241	0.2590
0.6657	0.0060	0.0361	0.0060	0.0241	0.2560
0.6687	0.0060	0.0361	0.0060	0.0241	0.2530
0.6717	0.0060	0.0090	0.0060	0.0241	0.2530
0.6747	0.0060	0.0090	0.0060	0.0241	0.2500
0.6777	0.0060	0.0090	0.0060	0.0241	0.2470
0.6807	0.0060	0.0090	0.0060	0.0241	0.2199
0.6837	0.0060	0.0090	0.0060	0.0241	0.2169
0.6867	0.0060	0.0090	0.0060	0.0241	0.2169
0.6898	0.0060	0.0090	0.0060	0.0241	0.2139
0.6928	0.0060	0.0090	0.0060	0.0241	0.2108
0.6958	0.0060	0.0090	0.0060	0.0241	0.2078
0.6988	0.0060	0.0060	0.0060	0.0241	0.2048
0.7018	0.0060	0.0060	0.0060	0.0211	0.2018
0.7048	0.0060	0.0060	0.0060	0.0211	0.1958
0.7078	0.0060	0.0060	0.0060	0.0181	0.1928
0.7108	0.0060	0.0060	0.0060	0.0181	0.1898
0.7139	0.0060	0.0060	0.0060	0.0151	0.1867
0.7169	0.0060	0.0060	0.0060	0.0151	0.1867

Removed _Ratio	GraphSAGE _Targeted_Attack	GAT _Targeted_Attack	GCN _Targeted_Attack	S_value _Targeted_attack	Random _Attack
0.7199	0.0030	0.0060	0.0060	0.0151	0.1807
0.7229	0.0030	0.0060	0.0060	0.0120	0.1777
0.7259	0.0030	0.0060	0.0060	0.0120	0.1717
0.7289	0.0030	0.0060	0.0060	0.0120	0.1717
0.7319	0.0030	0.0060	0.0060	0.0120	0.1687
0.7349	0.0030	0.0060	0.0060	0.0120	0.1657
0.7380	0.0030	0.0060	0.0060	0.0120	0.1627
0.7410	0.0030	0.0060	0.0060	0.0120	0.1596
0.7440	0.0030	0.0060	0.0060	0.0120	0.1566
0.7470	0.0030	0.0060	0.0060	0.0120	0.1536
0.7500	0.0030	0.0060	0.0060	0.0120	0.1506
0.7530	0.0030	0.0060	0.0060	0.0120	0.1476
0.7560	0.0030	0.0060	0.0060	0.0120	0.1446
0.7590	0.0030	0.0060	0.0060	0.0120	0.1446
0.7620	0.0030	0.0060	0.0060	0.0120	0.1416
0.7651	0.0030	0.0060	0.0060	0.0120	0.1416
0.7681	0.0030	0.0060	0.0060	0.0120	0.1416
0.7711	0.0030	0.0060	0.0060	0.0120	0.1386
0.7741	0.0030	0.0060	0.0060	0.0120	0.1386
0.7771	0.0030	0.0060	0.0060	0.0120	0.1386
0.7801	0.0030	0.0060	0.0060	0.0120	0.1386
0.7831	0.0030	0.0060	0.0060	0.0120	0.1355
0.7861	0.0030	0.0060	0.0060	0.0120	0.1325
0.7892	0.0030	0.0060	0.0060	0.0120	0.1295
0.7922	0.0030	0.0060	0.0060	0.0120	0.1265
0.7952	0.0030	0.0060	0.0060	0.0090	0.1265
0.7982	0.0030	0.0060	0.0060	0.0090	0.1265
0.8012	0.0030	0.0060	0.0060	0.0090	0.1114
0.8042	0.0030	0.0060	0.0060	0.0090	0.1114
0.8072	0.0030	0.0060	0.0060	0.0090	0.0904
0.8102	0.0030	0.0060	0.0060	0.0090	0.0904
0.8133	0.0030	0.0060	0.0060	0.0090	0.0873
0.8163	0.0030	0.0060	0.0060	0.0090	0.0843
0.8193	0.0030	0.0060	0.0060	0.0090	0.0843
0.8223	0.0030	0.0060	0.0060	0.0090	0.0843
0.8253	0.0030	0.0060	0.0060	0.0090	0.0813
0.8283	0.0030	0.0060	0.0060	0.0090	0.0753
0.8313	0.0030	0.0060	0.0060	0.0090	0.0753
0.8343	0.0030	0.0060	0.0060	0.0090	0.0723
0.8373	0.0030	0.0060	0.0060	0.0090	0.0723

Removed _Ratio	GraphSAGE _Targeted_Attack	GAT _Targeted_Attack	GCN _Targeted_Attack	S_value _Targeted_attack	Random _Attack
0.8404	0.0030	0.0060	0.0060	0.0090	0.0663
0.8434	0.0030	0.0060	0.0060	0.0090	0.0633
0.8464	0.0030	0.0060	0.0060	0.0090	0.0602
0.8494	0.0030	0.0060	0.0060	0.0090	0.0572
0.8524	0.0030	0.0060	0.0060	0.0090	0.0542
0.8554	0.0030	0.0060	0.0060	0.0090	0.0542
0.8584	0.0030	0.0060	0.0060	0.0090	0.0482
0.8614	0.0030	0.0060	0.0060	0.0060	0.0482
0.8645	0.0030	0.0060	0.0060	0.0060	0.0452
0.8675	0.0030	0.0060	0.0060	0.0060	0.0452
0.8705	0.0030	0.0060	0.0060	0.0060	0.0452
0.8735	0.0030	0.0060	0.0030	0.0060	0.0452
0.8765	0.0030	0.0060	0.0030	0.0060	0.0422
0.8795	0.0030	0.0060	0.0030	0.0060	0.0392
0.8825	0.0030	0.0030	0.0030	0.0060	0.0331
0.8855	0.0030	0.0030	0.0030	0.0060	0.0331
0.8886	0.0030	0.0030	0.0030	0.0060	0.0331
0.8916	0.0030	0.0030	0.0030	0.0060	0.0301
0.8946	0.0030	0.0030	0.0030	0.0060	0.0301
0.8976	0.0030	0.0030	0.0030	0.0060	0.0271
0.9006	0.0030	0.0030	0.0030	0.0060	0.0241
0.9036	0.0030	0.0030	0.0030	0.0060	0.0241
0.9066	0.0030	0.0030	0.0030	0.0060	0.0241
0.9096	0.0030	0.0030	0.0030	0.0060	0.0241
0.9127	0.0030	0.0030	0.0030	0.0060	0.0241
0.9157	0.0030	0.0030	0.0030	0.0060	0.0211
0.9187	0.0030	0.0030	0.0030	0.0060	0.0211
0.9217	0.0030	0.0030	0.0030	0.0060	0.0211
0.9247	0.0030	0.0030	0.0030	0.0060	0.0211
0.9277	0.0030	0.0030	0.0030	0.0060	0.0211
0.9307	0.0030	0.0030	0.0030	0.0060	0.0211
0.9337	0.0030	0.0030	0.0030	0.0060	0.0211
0.9367	0.0030	0.0030	0.0030	0.0060	0.0211
0.9398	0.0030	0.0030	0.0030	0.0060	0.0181
0.9428	0.0030	0.0030	0.0030	0.0060	0.0151
0.9458	0.0030	0.0030	0.0030	0.0060	0.0151
0.9488	0.0030	0.0030	0.0030	0.0060	0.0060
0.9518	0.0030	0.0030	0.0030	0.0060	0.0060
0.9548	0.0030	0.0030	0.0030	0.0060	0.0060
0.9578	0.0030	0.0030	0.0030	0.0060	0.0060

Removed _Ratio	GraphSAGE _Targeted_Attack	GAT _Targeted_Attack	GCN _Targeted_Attack	S_value _Targeted_attack	Random _Attack
0.9608	0.0030	0.0030	0.0030	0.0060	0.0060
0.9639	0.0030	0.0030	0.0030	0.0060	0.0060
0.9669	0.0030	0.0030	0.0030	0.0060	0.0060
0.9699	0.0030	0.0030	0.0030	0.0060	0.0060
0.9729	0.0030	0.0030	0.0030	0.0060	0.0060
0.9759	0.0030	0.0030	0.0030	0.0060	0.0060
0.9789	0.0030	0.0030	0.0030	0.0060	0.0030
0.9819	0.0030	0.0030	0.0030	0.0030	0.0030
0.9849	0.0030	0.0030	0.0030	0.0030	0.0030
0.9880	0.0030	0.0030	0.0030	0.0030	0.0030
0.9910	0.0030	0.0030	0.0030	0.0030	0.0030
0.9940	0.0030	0.0030	0.0030	0.0030	0.0030
0.9970	0.0030	0.0030	0.0030	0.0030	0.0030
1.0000	0.0000	0.0000	0.0000	0.0000	0.0000

Table S7. Efficiency——Targeted vs. Random Attacks: GraphSAGE versus Baseline Methods

Removed _Ratio	GraphSAGE _Targeted_Attack	GAT _Targeted_Attack	GCN _Targeted_Attack	S_value _Targeted_attack	Random _Attack
0.0030	0.3707	0.4061	0.3885	0.3707	0.4058
0.0060	0.3529	0.3886	0.3529	0.3529	0.4059
0.0090	0.3312	0.3811	0.3376	0.3312	0.4068
0.0120	0.2910	0.3803	0.3283	0.3155	0.4070
0.0151	0.2758	0.3639	0.3124	0.3058	0.4064
0.0181	0.2695	0.3627	0.2901	0.2901	0.4056
0.0211	0.2597	0.3441	0.2835	0.2511	0.4058
0.0241	0.2461	0.3280	0.2683	0.2446	0.4061
0.0271	0.2314	0.3250	0.2543	0.2314	0.4061
0.0301	0.2175	0.3242	0.2461	0.2175	0.4064
0.0331	0.2179	0.3186	0.2443	0.1990	0.4067
0.0361	0.1993	0.3121	0.2337	0.1912	0.4071
0.0392	0.1914	0.3110	0.2257	0.1885	0.4073
0.0422	0.1813	0.2628	0.2227	0.1887	0.4083
0.0452	0.1727	0.2601	0.2151	0.1802	0.4074
0.0482	0.1640	0.2582	0.2083	0.1699	0.4070
0.0512	0.1568	0.2385	0.1976	0.1610	0.4072
0.0542	0.1538	0.2295	0.1819	0.1538	0.4074
0.0572	0.1519	0.2200	0.1636	0.1519	0.4076
0.0602	0.1366	0.2137	0.1308	0.1458	0.4075
0.0633	0.1349	0.2126	0.1286	0.1440	0.4073
0.0663	0.1289	0.2035	0.1235	0.1390	0.4025
0.0693	0.1155	0.1978	0.1185	0.1337	0.3983
0.0723	0.1125	0.1975	0.1158	0.1325	0.3982
0.0753	0.1075	0.1962	0.1137	0.1176	0.3981
0.0783	0.1027	0.1937	0.1134	0.1047	0.3979
0.0813	0.0990	0.1891	0.1091	0.1029	0.3972
0.0843	0.0967	0.1889	0.1075	0.0989	0.3968
0.0873	0.0953	0.1848	0.1035	0.0972	0.3973
0.0904	0.0910	0.1844	0.1013	0.0937	0.3974
0.0934	0.0867	0.1841	0.0890	0.0898	0.3976
0.0964	0.0845	0.1821	0.0865	0.0884	0.3985
0.0994	0.0808	0.1810	0.0819	0.0845	0.3874
0.1024	0.0782	0.1707	0.0805	0.0818	0.3872
0.1054	0.0769	0.1694	0.0746	0.0790	0.3874
0.1084	0.0748	0.1691	0.0725	0.0775	0.3875
0.1114	0.0719	0.1678	0.0676	0.0769	0.3868
0.1145	0.0719	0.1672	0.0651	0.0708	0.3866
0.1175	0.0664	0.1665	0.0526	0.0712	0.3868

Removed _Ratio	GraphSAGE _Targeted_Attack	GAT _Targeted_Attack	GCN _Targeted_Attack	S_value _Targeted_attack	Random _Attack
0.1205	0.0615	0.1660	0.0503	0.0683	0.3867
0.1235	0.0523	0.1656	0.0476	0.0685	0.3871
0.1265	0.0495	0.1638	0.0474	0.0688	0.3874
0.1295	0.0470	0.1637	0.0394	0.0673	0.3876
0.1325	0.0444	0.1629	0.0394	0.0638	0.3869
0.1355	0.0419	0.1623	0.0377	0.0615	0.3870
0.1386	0.0421	0.1581	0.0351	0.0618	0.3876
0.1416	0.0342	0.1402	0.0335	0.0613	0.3871
0.1446	0.0287	0.1336	0.0286	0.0579	0.3873
0.1476	0.0272	0.1331	0.0221	0.0583	0.3865
0.1506	0.0246	0.1262	0.0217	0.0564	0.3865
0.1536	0.0226	0.1196	0.0213	0.0551	0.3865
0.1566	0.0222	0.1158	0.0206	0.0545	0.3865
0.1596	0.0212	0.1153	0.0206	0.0543	0.3866
0.1627	0.0169	0.1137	0.0203	0.0525	0.3871
0.1657	0.0166	0.1119	0.0191	0.0508	0.3838
0.1687	0.0159	0.1115	0.0191	0.0511	0.3815
0.1717	0.0150	0.1105	0.0181	0.0497	0.3794
0.1747	0.0148	0.1092	0.0181	0.0363	0.3794
0.1777	0.0148	0.0944	0.0151	0.0229	0.3792
0.1807	0.0147	0.0811	0.0147	0.0229	0.3788
0.1837	0.0140	0.0803	0.0144	0.0228	0.3794
0.1867	0.0111	0.0806	0.0143	0.0229	0.3779
0.1898	0.0107	0.0730	0.0142	0.0218	0.3781
0.1928	0.0106	0.0734	0.0133	0.0219	0.3785
0.1958	0.0104	0.0734	0.0132	0.0219	0.3783
0.1988	0.0093	0.0737	0.0132	0.0220	0.3786
0.2018	0.0093	0.0735	0.0104	0.0221	0.3785
0.2048	0.0094	0.0542	0.0100	0.0218	0.3792
0.2078	0.0092	0.0544	0.0099	0.0216	0.3800
0.2108	0.0091	0.0537	0.0098	0.0193	0.3802
0.2139	0.0088	0.0541	0.0094	0.0194	0.3806
0.2169	0.0087	0.0501	0.0088	0.0187	0.3808
0.2199	0.0081	0.0481	0.0079	0.0188	0.3808
0.2229	0.0078	0.0484	0.0079	0.0187	0.3809
0.2259	0.0077	0.0487	0.0077	0.0185	0.3814
0.2289	0.0075	0.0482	0.0077	0.0186	0.3814
0.2319	0.0074	0.0481	0.0076	0.0182	0.3822
0.2349	0.0069	0.0485	0.0073	0.0162	0.3823
0.2380	0.0068	0.0257	0.0068	0.0162	0.3821
0.2410	0.0063	0.0259	0.0067	0.0161	0.3824

Removed _Ratio	GraphSAGE _Targeted_Attack	GAT _Targeted_Attack	GCN _Targeted_Attack	S_value _Targeted_attack	Random _Attack
0.2440	0.0063	0.0257	0.0067	0.0162	0.3826
0.2470	0.0061	0.0256	0.0067	0.0155	0.3819
0.2500	0.0060	0.0257	0.0067	0.0139	0.3815
0.2530	0.0059	0.0256	0.0066	0.0140	0.3845
0.2560	0.0058	0.0253	0.0063	0.0134	0.3856
0.2590	0.0056	0.0255	0.0059	0.0133	0.3865
0.2620	0.0056	0.0257	0.0057	0.0126	0.3868
0.2651	0.0056	0.0257	0.0055	0.0127	0.3879
0.2681	0.0054	0.0255	0.0055	0.0124	0.3885
0.2711	0.0054	0.0257	0.0055	0.0121	0.3859
0.2741	0.0051	0.0257	0.0055	0.0116	0.3862
0.2771	0.0051	0.0259	0.0054	0.0114	0.3866
0.2801	0.0047	0.0259	0.0052	0.0099	0.3849
0.2831	0.0046	0.0258	0.0052	0.0098	0.3851
0.2861	0.0045	0.0257	0.0048	0.0089	0.3848
0.2892	0.0043	0.0259	0.0047	0.0088	0.3848
0.2922	0.0043	0.0259	0.0046	0.0088	0.3846
0.2952	0.0041	0.0259	0.0046	0.0088	0.3838
0.2982	0.0042	0.0257	0.0046	0.0088	0.3841
0.3012	0.0041	0.0257	0.0046	0.0088	0.3729
0.3042	0.0039	0.0259	0.0046	0.0087	0.3734
0.3072	0.0039	0.0261	0.0044	0.0085	0.3728
0.3102	0.0038	0.0255	0.0043	0.0084	0.3707
0.3133	0.0038	0.0255	0.0044	0.0084	0.3695
0.3163	0.0037	0.0256	0.0043	0.0084	0.3690
0.3193	0.0037	0.0256	0.0043	0.0084	0.3688
0.3223	0.0037	0.0258	0.0042	0.0080	0.3689
0.3253	0.0037	0.0259	0.0041	0.0080	0.3687
0.3283	0.0037	0.0261	0.0039	0.0078	0.3678
0.3313	0.0036	0.0264	0.0038	0.0077	0.3683
0.3343	0.0036	0.0266	0.0036	0.0078	0.3673
0.3373	0.0036	0.0269	0.0034	0.0076	0.3707
0.3404	0.0036	0.0270	0.0034	0.0075	0.3516
0.3434	0.0034	0.0272	0.0033	0.0075	0.3517
0.3464	0.0033	0.0271	0.0033	0.0069	0.3549
0.3494	0.0033	0.0272	0.0032	0.0064	0.3549
0.3524	0.0032	0.0265	0.0032	0.0058	0.3530
0.3554	0.0032	0.0268	0.0032	0.0058	0.3525
0.3584	0.0033	0.0270	0.0032	0.0058	0.3515
0.3614	0.0032	0.0272	0.0032	0.0053	0.3513
0.3645	0.0030	0.0274	0.0030	0.0052	0.3502

Removed _Ratio	GraphSAGE _Targeted_Attack	GAT _Targeted_Attack	GCN _Targeted_Attack	S_value _Targeted_attack	Random _Attack
0.3675	0.0030	0.0276	0.0031	0.0052	0.3499
0.3705	0.0029	0.0278	0.0031	0.0052	0.3532
0.3735	0.0028	0.0272	0.0031	0.0041	0.3525
0.3765	0.0028	0.0266	0.0031	0.0039	0.3519
0.3795	0.0028	0.0268	0.0030	0.0038	0.3515
0.3825	0.0028	0.0268	0.0030	0.0037	0.3525
0.3855	0.0026	0.0270	0.0029	0.0037	0.3516
0.3886	0.0026	0.0273	0.0028	0.0037	0.3517
0.3916	0.0026	0.0275	0.0029	0.0027	0.3514
0.3946	0.0025	0.0278	0.0029	0.0027	0.3512
0.3976	0.0024	0.0278	0.0029	0.0027	0.3522
0.4006	0.0023	0.0281	0.0029	0.0025	0.3523
0.4036	0.0023	0.0283	0.0029	0.0023	0.3457
0.4066	0.0023	0.0286	0.0028	0.0022	0.3189
0.4096	0.0024	0.0281	0.0026	0.0022	0.3163
0.4127	0.0024	0.0284	0.0024	0.0022	0.3160
0.4157	0.0024	0.0206	0.0024	0.0022	0.3193
0.4187	0.0024	0.0208	0.0022	0.0022	0.3224
0.4217	0.0022	0.0209	0.0022	0.0022	0.3236
0.4247	0.0020	0.0211	0.0021	0.0022	0.3232
0.4277	0.0021	0.0213	0.0021	0.0022	0.3266
0.4307	0.0020	0.0215	0.0021	0.0022	0.3065
0.4337	0.0020	0.0211	0.0020	0.0022	0.3055
0.4367	0.0019	0.0212	0.0020	0.0022	0.3053
0.4398	0.0018	0.0214	0.0020	0.0023	0.3085
0.4428	0.0018	0.0216	0.0019	0.0022	0.3082
0.4458	0.0017	0.0218	0.0020	0.0023	0.3080
0.4488	0.0017	0.0220	0.0020	0.0023	0.3079
0.4518	0.0017	0.0222	0.0019	0.0023	0.3113
0.4548	0.0017	0.0224	0.0018	0.0022	0.3106
0.4578	0.0016	0.0227	0.0018	0.0022	0.3140
0.4608	0.0016	0.0228	0.0018	0.0022	0.3135
0.4639	0.0015	0.0229	0.0019	0.0023	0.3132
0.4669	0.0013	0.0232	0.0019	0.0023	0.3146
0.4699	0.0014	0.0221	0.0019	0.0022	0.3126
0.4729	0.0013	0.0217	0.0019	0.0022	0.3115
0.4759	0.0013	0.0218	0.0019	0.0022	0.3110
0.4789	0.0013	0.0220	0.0019	0.0022	0.3102
0.4819	0.0013	0.0222	0.0019	0.0022	0.3100
0.4849	0.0012	0.0222	0.0019	0.0022	0.3107
0.4880	0.0013	0.0224	0.0018	0.0023	0.3116

Removed _Ratio	GraphSAGE _Targeted_Attack	GAT _Targeted_Attack	GCN _Targeted_Attack	S_value _Targeted_attack	Random _Attack
0.4910	0.0013	0.0219	0.0016	0.0023	0.3108
0.4940	0.0013	0.0221	0.0015	0.0022	0.3022
0.4970	0.0013	0.0223	0.0015	0.0023	0.3006
0.5000	0.0012	0.0224	0.0014	0.0023	0.2998
0.5030	0.0013	0.0217	0.0014	0.0023	0.2995
0.5060	0.0012	0.0208	0.0015	0.0024	0.2988
0.5090	0.0011	0.0199	0.0013	0.0024	0.2976
0.5120	0.0012	0.0192	0.0013	0.0024	0.2973
0.5151	0.0012	0.0195	0.0013	0.0024	0.2882
0.5181	0.0012	0.0197	0.0013	0.0025	0.2878
0.5211	0.0012	0.0200	0.0013	0.0025	0.2874
0.5241	0.0012	0.0201	0.0013	0.0025	0.2911
0.5271	0.0011	0.0204	0.0012	0.0026	0.2906
0.5301	0.0012	0.0195	0.0012	0.0026	0.2904
0.5331	0.0012	0.0187	0.0012	0.0026	0.2901
0.5361	0.0012	0.0180	0.0012	0.0027	0.2938
0.5392	0.0011	0.0181	0.0012	0.0027	0.2942
0.5422	0.0011	0.0184	0.0011	0.0027	0.2938
0.5452	0.0011	0.0186	0.0011	0.0028	0.2934
0.5482	0.0012	0.0189	0.0012	0.0027	0.2942
0.5512	0.0011	0.0191	0.0011	0.0028	0.2934
0.5542	0.0011	0.0194	0.0011	0.0028	0.2921
0.5572	0.0011	0.0184	0.0011	0.0027	0.2909
0.5602	0.0011	0.0175	0.0011	0.0028	0.2888
0.5633	0.0011	0.0177	0.0011	0.0028	0.2883
0.5663	0.0012	0.0169	0.0011	0.0029	0.2924
0.5693	0.0012	0.0166	0.0011	0.0029	0.2816
0.5723	0.0011	0.0169	0.0011	0.0028	0.2811
0.5753	0.0011	0.0169	0.0011	0.0029	0.2851
0.5783	0.0011	0.0171	0.0010	0.0029	0.2844
0.5813	0.0011	0.0164	0.0010	0.0030	0.2809
0.5843	0.0011	0.0157	0.0010	0.0030	0.2848
0.5873	0.0011	0.0159	0.0010	0.0031	0.2843
0.5904	0.0011	0.0161	0.0009	0.0031	0.2886
0.5934	0.0011	0.0153	0.0009	0.0032	0.2928
0.5964	0.0010	0.0155	0.0009	0.0029	0.2932
0.5994	0.0010	0.0158	0.0009	0.0030	0.2920
0.6024	0.0010	0.0159	0.0009	0.0030	0.2925
0.6054	0.0009	0.0107	0.0009	0.0031	0.2969
0.6084	0.0008	0.0107	0.0010	0.0031	0.2966
0.6114	0.0008	0.0109	0.0010	0.0031	0.2958

Removed _Ratio	GraphSAGE _Targeted_Attack	GAT _Targeted_Attack	GCN _Targeted_Attack	S_value _Targeted_attack	Random _Attack
0.6145	0.0009	0.0111	0.0010	0.0032	0.2958
0.6175	0.0007	0.0111	0.0009	0.0031	0.2930
0.6205	0.0008	0.0113	0.0009	0.0032	0.2927
0.6235	0.0008	0.0095	0.0009	0.0032	0.2918
0.6265	0.0008	0.0087	0.0009	0.0033	0.2916
0.6295	0.0008	0.0089	0.0009	0.0032	0.2890
0.6325	0.0008	0.0090	0.0009	0.0033	0.2535
0.6355	0.0007	0.0090	0.0010	0.0033	0.2528
0.6386	0.0007	0.0092	0.0010	0.0034	0.2497
0.6416	0.0007	0.0093	0.0010	0.0034	0.2485
0.6446	0.0007	0.0085	0.0010	0.0035	0.2489
0.6476	0.0007	0.0086	0.0009	0.0035	0.2532
0.6506	0.0007	0.0086	0.0009	0.0036	0.2576
0.6536	0.0008	0.0088	0.0009	0.0037	0.2562
0.6566	0.0008	0.0078	0.0009	0.0037	0.2551
0.6596	0.0008	0.0079	0.0008	0.0038	0.2596
0.6627	0.0008	0.0080	0.0008	0.0039	0.2565
0.6657	0.0008	0.0082	0.0008	0.0039	0.2530
0.6687	0.0008	0.0082	0.0008	0.0040	0.2517
0.6717	0.0008	0.0023	0.0008	0.0041	0.2564
0.6747	0.0009	0.0023	0.0009	0.0042	0.2568
0.6777	0.0007	0.0024	0.0009	0.0042	0.2557
0.6807	0.0007	0.0024	0.0009	0.0043	0.2090
0.6837	0.0005	0.0025	0.0009	0.0044	0.2078
0.6867	0.0004	0.0025	0.0009	0.0045	0.2114
0.6898	0.0004	0.0024	0.0010	0.0046	0.2090
0.6928	0.0004	0.0024	0.0010	0.0047	0.2063
0.6958	0.0004	0.0025	0.0008	0.0048	0.2046
0.6988	0.0004	0.0020	0.0008	0.0048	0.2045
0.7018	0.0004	0.0021	0.0008	0.0042	0.2014
0.7048	0.0004	0.0021	0.0008	0.0043	0.1961
0.7078	0.0004	0.0021	0.0009	0.0034	0.1935
0.7108	0.0002	0.0022	0.0009	0.0035	0.1913
0.7139	0.0002	0.0022	0.0009	0.0029	0.1902
0.7169	0.0002	0.0023	0.0009	0.0030	0.1943
0.7199	0.0000	0.0023	0.0009	0.0030	0.1890
0.7229	0.0000	0.0024	0.0010	0.0023	0.1887
0.7259	0.0000	0.0024	0.0010	0.0023	0.1819
0.7289	0.0000	0.0025	0.0010	0.0024	0.1860
0.7319	0.0000	0.0026	0.0010	0.0024	0.1858
0.7349	0.0000	0.0024	0.0010	0.0025	0.1835

Removed _Ratio	GraphSAGE _Targeted_Attack	GAT _Targeted_Attack	GCN _Targeted_Attack	S_value _Targeted_attack	Random _Attack
0.7380	0.0000	0.0024	0.0011	0.0025	0.1806
0.7410	0.0000	0.0022	0.0011	0.0026	0.1804
0.7440	0.0000	0.0022	0.0011	0.0027	0.1764
0.7470	0.0000	0.0023	0.0011	0.0027	0.1731
0.7500	0.0000	0.0024	0.0012	0.0028	0.1720
0.7530	0.0000	0.0021	0.0012	0.0029	0.1706
0.7560	0.0000	0.0022	0.0009	0.0029	0.1683
0.7590	0.0000	0.0019	0.0009	0.0027	0.1726
0.7620	0.0000	0.0019	0.0010	0.0028	0.1702
0.7651	0.0000	0.0017	0.0010	0.0028	0.1746
0.7681	0.0000	0.0017	0.0010	0.0029	0.1792
0.7711	0.0000	0.0018	0.0007	0.0030	0.1779
0.7741	0.0000	0.0018	0.0007	0.0031	0.1823
0.7771	0.0000	0.0019	0.0007	0.0031	0.1873
0.7801	0.0000	0.0015	0.0008	0.0032	0.1925
0.7831	0.0000	0.0016	0.0004	0.0033	0.1897
0.7861	0.0000	0.0016	0.0004	0.0034	0.1881
0.7892	0.0000	0.0017	0.0004	0.0035	0.1830
0.7922	0.0000	0.0017	0.0004	0.0036	0.1775
0.7952	0.0000	0.0018	0.0004	0.0029	0.1828
0.7982	0.0000	0.0018	0.0005	0.0029	0.1879
0.8012	0.0000	0.0019	0.0005	0.0030	0.1432
0.8042	0.0000	0.0019	0.0005	0.0031	0.1472
0.8072	0.0000	0.0020	0.0005	0.0032	0.0985
0.8102	0.0000	0.0020	0.0005	0.0028	0.1017
0.8133	0.0000	0.0021	0.0005	0.0029	0.1003
0.8163	0.0000	0.0022	0.0005	0.0030	0.0984
0.8193	0.0000	0.0023	0.0006	0.0031	0.1018
0.8223	0.0000	0.0018	0.0006	0.0032	0.1047
0.8253	0.0000	0.0018	0.0006	0.0027	0.1017
0.8283	0.0000	0.0019	0.0006	0.0028	0.0937
0.8313	0.0000	0.0019	0.0006	0.0029	0.0971
0.8343	0.0000	0.0020	0.0007	0.0030	0.0904
0.8373	0.0000	0.0021	0.0007	0.0031	0.0938
0.8404	0.0000	0.0022	0.0007	0.0033	0.0853
0.8434	0.0000	0.0023	0.0008	0.0034	0.0819
0.8464	0.0000	0.0024	0.0008	0.0035	0.0801
0.8494	0.0000	0.0024	0.0008	0.0037	0.0747
0.8524	0.0000	0.0026	0.0009	0.0038	0.0716
0.8554	0.0000	0.0027	0.0009	0.0040	0.0746
0.8584	0.0000	0.0019	0.0009	0.0042	0.0614

Removed _Ratio	GraphSAGE _Targeted_Attack	GAT _Targeted_Attack	GCN _Targeted_Attack	S_value _Targeted_attack	Random _Attack
0.8614	0.0000	0.0019	0.0010	0.0019	0.0631
0.8645	0.0000	0.0020	0.0010	0.0020	0.0596
0.8675	0.0000	0.0021	0.0011	0.0021	0.0597
0.8705	0.0000	0.0011	0.0011	0.0022	0.0626
0.8735	0.0000	0.0012	0.0000	0.0023	0.0656
0.8765	0.0000	0.0012	0.0000	0.0024	0.0596
0.8795	0.0000	0.0013	0.0000	0.0026	0.0549
0.8825	0.0000	0.0000	0.0000	0.0027	0.0396
0.8855	0.0000	0.0000	0.0000	0.0028	0.0417
0.8886	0.0000	0.0000	0.0000	0.0030	0.0440
0.8916	0.0000	0.0000	0.0000	0.0032	0.0397
0.8946	0.0000	0.0000	0.0000	0.0034	0.0420
0.8976	0.0000	0.0000	0.0000	0.0036	0.0377
0.9006	0.0000	0.0000	0.0000	0.0038	0.0333
0.9036	0.0000	0.0000	0.0000	0.0040	0.0334
0.9066	0.0000	0.0000	0.0000	0.0043	0.0357
0.9096	0.0000	0.0000	0.0000	0.0046	0.0381
0.9127	0.0000	0.0000	0.0000	0.0049	0.0408
0.9157	0.0000	0.0000	0.0000	0.0053	0.0353
0.9187	0.0000	0.0000	0.0000	0.0057	0.0380
0.9217	0.0000	0.0000	0.0000	0.0062	0.0410
0.9247	0.0000	0.0000	0.0000	0.0067	0.0444
0.9277	0.0000	0.0000	0.0000	0.0072	0.0483
0.9307	0.0000	0.0000	0.0000	0.0079	0.0527
0.9337	0.0000	0.0000	0.0000	0.0087	0.0577
0.9367	0.0000	0.0000	0.0000	0.0048	0.0635
0.9398	0.0000	0.0000	0.0000	0.0053	0.0553
0.9428	0.0000	0.0000	0.0000	0.0058	0.0439
0.9458	0.0000	0.0000	0.0000	0.0065	0.0490
0.9488	0.0000	0.0000	0.0000	0.0074	0.0074
0.9518	0.0000	0.0000	0.0000	0.0083	0.0083
0.9548	0.0000	0.0000	0.0000	0.0095	0.0095
0.9578	0.0000	0.0000	0.0000	0.0110	0.0110
0.9608	0.0000	0.0000	0.0000	0.0128	0.0128
0.9639	0.0000	0.0000	0.0000	0.0152	0.0152
0.9669	0.0000	0.0000	0.0000	0.0182	0.0182
0.9699	0.0000	0.0000	0.0000	0.0222	0.0222
0.9729	0.0000	0.0000	0.0000	0.0278	0.0278
0.9759	0.0000	0.0000	0.0000	0.0357	0.0357
0.9789	0.0000	0.0000	0.0000	0.0476	0.0000
0.9819	0.0000	0.0000	0.0000	0.0000	0.0000

Removed<br>_Ratio	GraphSAGE<br>_Targeted_Attack	GAT<br>_Targeted_Attack	GCN<br>_Targeted_Attack	S_value<br>_Targeted_attack	Random<br>_Attack
0.9849	0.0000	0.0000	0.0000	0.0000	0.0000
0.9880	0.0000	0.0000	0.0000	0.0000	0.0000
0.9910	0.0000	0.0000	0.0000	0.0000	0.0000
0.9940	0.0000	0.0000	0.0000	0.0000	0.0000
0.9970	0.0000	0.0000	0.0000	0.0000	0.0000
1.0000	0.0000	0.0000	0.0000	0.0000	0.0000

Table S8. Critical node ranking: GraphSAGE vs Baselines

Node	GAT	GCN	GraphSAGE	S
1	169	188	191	137
2	168	187	190	136
3	251	252	228	139
4	214	164	117	62
5	250	251	229	138
6	186	291	243	141
7	185	290	239	140
8	223	20	4	7
9	125	107	116	151
10	124	106	105	46
11	141	167	184	152
12	142	168	185	153
13	138	26	11	14
14	224	324	289	154
15	225	325	290	155
16	208	225	237	142
17	158	144	152	156
18	226	326	291	157
19	159	145	166	158
20	160	146	167	159
21	227	327	292	160
22	167	183	148	128
23	259	260	234	143
24	260	261	227	144
25	253	233	270	161
26	207	50	38	53
27	276	332	332	145
28	242	249	251	146
29	194	140	162	162
30	254	256	225	147
31	170	108	130	98
32	144	114	115	131
33	193	126	88	99
34	255	257	235	148
35	218	163	145	96
36	233	185	170	149
37	249	254	256	150
38	232	169	140	97
39	240	259	268	163

Node	GAT	GCN	GraphSAGE	S
167	51	34	35	32
168	238	311	249	229
169	70	133	118	175
170	24	217	182	213
171	58	75	91	126
172	82	52	47	130
173	264	331	274	230
174	20	22	25	22
175	295	289	316	261
176	6	21	24	31
177	47	23	26	23
178	315	278	307	266
179	10	37	40	59
180	304	298	313	267
181	266	203	178	114
182	7	6	3	3
183	72	57	53	50
184	258	330	273	262
185	316	279	308	277
186	204	268	246	231
187	309	285	304	255
188	153	194	169	268
189	96	123	81	79
190	182	255	255	265
191	181	306	248	232
192	21	112	89	89
193	317	280	309	278
194	297	237	281	279
195	274	173	198	269
196	305	299	314	270
197	39	85	78	85
198	119	160	137	233
199	281	247	262	280
200	283	213	261	281
201	68	8	10	10
202	115	88	86	51
203	33	61	56	43
204	133	198	159	234
205	282	248	263	282

Node	GAT	GCN	GraphSAGE	S
40	56	74	109	164
41	212	226	247	166
42	291	301	319	167
43	311	272	310	168
44	312	273	311	169
45	140	39	41	63
46	76	83	102	64
47	79	13	12	11
48	203	212	250	171
49	148	101	111	102
50	104	65	90	65
51	199	276	260	177
52	313	274	312	178
53	179	175	179	100
54	89	117	153	170
55	53	63	94	66
56	134	99	136	172
57	154	89	92	104
58	81	92	85	129
59	103	62	72	101
60	106	53	60	103
61	36	76	139	165
62	105	66	75	67
63	108	81	84	61
64	164	171	232	181
65	73	18	20	25
66	162	82	134	182
67	17	9	8	9
68	30	139	131	194
69	183	104	129	179
70	192	208	230	180
71	99	51	52	83
72	129	224	193	196
73	64	84	77	95
74	206	201	220	173
75	189	58	59	39
76	235	127	138	185
77	118	159	177	195
78	229	215	223	186
79	230	216	211	105
80	71	153	149	80

Node	GAT	GCN	GraphSAGE	S
206	143	136	128	120
207	273	174	203	271
208	318	243	293	285
209	247	220	245	272
210	310	286	305	256
211	306	300	315	273
212	135	132	112	214
213	32	47	43	35
214	279	307	288	283
215	271	329	276	257
216	100	97	83	235
217	15	24	21	29
218	132	79	80	90
219	27	33	30	44
220	244	231	236	274
221	9	14	18	13
222	184	236	199	236
223	277	305	285	289
224	180	238	221	276
225	155	134	126	237
226	245	232	238	275
227	302	262	298	290
228	285	205	183	122
229	74	121	100	115
230	8	5	9	6
231	319	244	294	298
232	23	29	27	33
233	122	54	55	107
234	272	304	284	292
235	210	240	258	288
236	286	206	217	284
237	217	293	259	249
238	303	263	299	293
239	113	152	142	264
240	171	258	215	238
241	321	312	320	301
242	150	151	155	72
243	257	295	267	259
244	296	246	296	302
245	44	105	96	86
246	61	86	65	87

Node	GAT	GCN	GraphSAGE	S
81	231	269	233	199
82	117	64	57	93
83	165	42	33	82
84	228	302	283	202
85	127	143	141	198
86	187	287	272	174
87	161	130	195	183
88	298	281	317	203
89	284	239	278	204
90	219	189	174	187
91	41	119	108	206
92	40	102	95	205
93	151	227	218	207
94	59	43	48	124
95	38	93	76	37
96	131	158	161	94
97	93	110	157	201
98	174	190	242	197
99	92	242	200	188
100	91	115	110	109
101	80	111	101	84
102	191	147	143	210
103	290	197	206	211
104	145	170	165	127
105	147	275	257	212
106	97	162	158	200
107	215	195	207	209
108	190	182	212	184
109	42	30	32	40
110	241	296	277	216
111	173	184	171	189
112	12	12	16	17
113	268	292	275	220
114	299	282	318	221
115	289	196	194	111
116	98	56	82	42
117	307	283	302	222
118	2	1	2	2
119	49	70	64	68
120	221	137	124	133
121	288	207	271	241

Node	GAT	GCN	GraphSAGE	S
247	322	313	321	303
248	34	19	15	15
249	175	129	147	135
250	45	165	133	92
251	116	221	186	263
252	267	308	264	258
253	111	103	99	176
254	28	122	125	291
255	3	4	7	5
256	52	73	61	54
257	323	314	322	308
258	19	17	14	16
259	149	218	208	294
260	84	148	146	239
261	14	2	1	1
262	1	49	66	286
263	57	46	44	78
264	320	245	295	309
265	300	265	300	305
266	62	80	79	75
267	69	98	104	306
268	324	315	323	310
269	294	192	209	311
270	293	193	202	312
271	43	94	103	121
272	109	149	172	295
273	114	71	71	55
274	130	161	163	193
275	31	157	151	300
276	88	78	74	240
277	325	316	324	313
278	326	317	325	314
279	327	318	326	315
280	328	319	327	316
281	90	87	98	123
282	329	320	328	317
283	101	96	113	74
284	94	67	62	91
285	280	235	280	299
286	77	142	156	287
287	102	294	254	296

Node	GAT	GCN	GraphSAGE	S
122	216	131	173	242
123	50	48	51	48
124	278	181	231	244
125	137	109	114	108
126	87	229	188	217
127	234	267	252	223
128	239	253	214	190
129	213	270	240	208
130	163	72	73	70
131	25	36	36	36
132	275	328	266	246
133	126	141	121	191
134	252	219	222	224
135	256	154	226	247
136	292	223	205	106
137	85	95	93	110
138	211	166	160	118
139	200	118	97	77
140	63	186	150	134
141	146	210	224	248
142	29	41	42	58
143	78	125	122	47
144	22	10	13	12
145	172	209	181	225
146	4	27	31	28
147	16	11	19	19
148	166	135	144	112
149	248	250	219	218
150	35	32	34	30
151	196	113	120	56
152	5	3	5	4
153	139	264	216	192
154	209	124	107	113
155	243	179	176	226
156	308	284	303	254
157	246	128	132	71
158	195	155	106	227
159	110	90	68	52
160	237	230	253	245
161	46	35	39	38
162	18	16	22	20

Node	GAT	GCN	GraphSAGE	S
288	152	60	69	116
289	301	266	301	307
290	262	310	287	318
291	330	321	329	319
292	120	59	54	125
293	13	15	17	18
294	331	322	330	320
295	270	303	282	297
296	54	40	45	45
297	128	91	87	117
298	112	211	204	119
299	26	25	28	27
300	65	180	175	250
301	37	38	37	34
302	86	241	241	243
303	198	288	265	321
304	332	323	331	322
305	121	138	119	219
306	55	77	67	215
307	75	68	63	69
308	67	234	210	323
309	261	309	286	324
310	60	45	50	57
311	48	28	29	24
312	176	199	192	325
313	201	31	23	26
314	197	172	187	326
315	202	177	196	76
316	222	44	58	41
317	188	100	123	327
318	236	116	154	73
319	177	200	197	328
320	83	204	213	251
321	123	55	49	21
322	95	150	168	252
323	265	222	269	330
324	107	176	201	81
325	66	156	180	253
326	287	271	297	329
327	156	69	70	60
328	136	202	135	132

Node	GAT	GCN	GraphSAGE	S
163	269	178	127	88
164	205	228	244	228
165	314	277	306	260
166	11	7	6	8

Node	GAT	GCN	GraphSAGE	S
329	178	120	46	49
330	157	191	164	331
331	263	297	279	304
332	220	214	189	332

Table S9. Jaccard similarity coefficient:GraphSAGE vs. Baseline Methods for Critical Node Ranking

	GraphSAGE	S	DC	CC	EC	BC	H
**GraphSAGE**	1.00	0.78	0.69	0.69	0.65	0.69	0.65
**S**	0.78	1.00	0.74	0.69	0.69	0.61	0.74
**DC**	0.69	0.74	1.00	0.78	0.74	0.47	0.94
**CC**	0.69	0.69	0.78	1.00	0.78	0.47	0.74
**EC**	0.65	0.69	0.74	0.78	1.00	0.47	0.74
**BC**	0.69	0.61	0.47	0.47	0.47	1.00	0.43
**H**	0.65	0.74	0.94	0.74	0.74	0.43	1.00

Table S10. Resilience metric weight fluctuation sample(200)

	w_survival	w_critical	w_stability		w_survival	w_critical	w_stability
**1**	0.50154475	0.303363201	0.195092049	**101**	0.466829572	0.327986123	0.205184305
**2**	0.493082144	0.310587725	0.196330131	**102**	0.452142958	0.301413578	0.246443465
**3**	0.501962287	0.281671836	0.216365877	**103**	0.492739608	0.277042354	0.230218038
**4**	0.495949028	0.322068537	0.181982435	**104**	0.503585361	0.315099127	0.181315511
**5**	0.50303988	0.297446887	0.199513233	**105**	0.498694986	0.266475433	0.234829581
**6**	0.446749754	0.323691268	0.229558978	**106**	0.500724215	0.300215159	0.199060627
**7**	0.490227729	0.327278591	0.18249368	**107**	0.529542601	0.297249428	0.173207971
**8**	0.469296529	0.272104396	0.258599075	**108**	0.5006812	0.274942228	0.224376572
**9**	0.489343	0.306367654	0.204289345	**109**	0.533886911	0.260139655	0.205973435
**10**	0.516347804	0.307682227	0.175969969	**110**	0.458059558	0.324483039	0.217457404
**11**	0.525661062	0.311156574	0.163182364	**111**	0.4801308	0.338628361	0.18124084
**12**	0.503653489	0.304275536	0.192070975	**112**	0.510357767	0.27824832	0.211393913
**13**	0.528896553	0.248954328	0.222149119	**113**	0.472798815	0.334810885	0.1923903
**14**	0.454186323	0.330848804	0.214964873	**114**	0.487356772	0.341269685	0.171373544
**15**	0.47740433	0.345151259	0.177444411	**115**	0.514860773	0.283507291	0.201631937
**16**	0.51217491	0.329765491	0.158059599	**116**	0.476648796	0.308671488	0.214679716
**17**	0.481253529	0.279616392	0.239130079	**117**	0.518269203	0.282835809	0.198894989
**18**	0.49224718	0.281724648	0.226028172	**118**	0.490855154	0.275996794	0.233148052
**19**	0.484737754	0.27644227	0.238819976	**119**	0.48540114	0.323724485	0.190874375

**20**	0.493488256	0.331688905	0.174822839	**120**	0.450346919	0.329570257	0.220082824
**21**	0.525093751	0.263436931	0.211469319	**121**	0.497666623	0.331919537	0.17041384
**22**	0.536453734	0.313436075	0.150110191	**122**	0.521158998	0.302328006	0.176512996
**23**	0.511725759	0.279526564	0.208747677	**123**	0.515682854	0.298041882	0.186275264
**24**	0.519431601	0.302854791	0.177713607	**124**	0.524938256	0.307095133	0.16796661
**25**	0.488377133	0.295401346	0.21622152	**125**	0.555065397	0.288673188	0.156261415
**26**	0.504391639	0.294941273	0.200667088	**126**	0.522991076	0.284681123	0.192327802
**27**	0.512839419	0.2756984	0.211462182	**127**	0.522596594	0.27756865	0.199834756
**28**	0.49646796	0.293785758	0.209746282	**128**	0.499924744	0.331876651	0.168198604
**29**	0.508016295	0.315256386	0.176727319	**129**	0.469386753	0.325136991	0.205476256
**30**	0.520106526	0.301520373	0.178373101	**130**	0.532234962	0.306573449	0.16119159
**31**	0.498127859	0.311983414	0.189888727	**131**	0.517130961	0.319742515	0.163126525
**32**	0.478036406	0.32336617	0.198597424	**132**	0.477654635	0.322514171	0.199831194
**33**	0.501867395	0.280900354	0.21723225	**133**	0.522433388	0.282696201	0.194870411
**34**	0.508813389	0.299109885	0.192076726	**134**	0.464477544	0.31670817	0.218814286
**35**	0.474680854	0.31966389	0.205655256	**135**	0.483984275	0.32291797	0.193097755
**36**	0.521236427	0.258160833	0.22060274	**136**	0.535115178	0.253202999	0.211681823
**37**	0.448851075	0.318235424	0.232913501	**137**	0.507126578	0.319602013	0.173271409
**38**	0.50801625	0.272483428	0.219500323	**138**	0.511498764	0.285814132	0.202687104
**39**	0.491688222	0.279308526	0.229003252	**139**	0.523929874	0.247621709	0.228448417
**40**	0.502116137	0.324982768	0.172901096	**140**	0.463733343	0.3401189	0.196147758
**41**	0.534269946	0.251779726	0.213950328	**141**	0.48633401	0.344134009	0.169531981
**42**	0.502380057	0.328984908	0.168635035	**142**	0.512276256	0.303608712	0.184115032
**43**	0.540324914	0.288705384	0.170969702	**143**	0.482251824	0.355711311	0.162036865
**44**	0.498241928	0.30724462	0.194513453	**144**	0.495505646	0.264971624	0.23952273
**45**	0.523095952	0.287535961	0.189368087	**145**	0.501085866	0.305516395	0.193397739
**46**	0.493674124	0.34179356	0.164532317	**146**	0.479358473	0.274079932	0.246561596
**47**	0.479923805	0.277008927	0.243067268	**147**	0.559223978	0.275472813	0.165303209
**48**	0.476629642	0.275344308	0.24802605	**148**	0.508453932	0.3170054	0.174540668
**49**	0.490591646	0.27958281	0.229825544	**149**	0.488566709	0.309055027	0.202378264
**50**	0.494627259	0.33365947	0.17171327	**150**	0.470233315	0.309241289	0.220525396
**51**	0.47332152	0.298732256	0.227946224	**151**	0.516483853	0.323696657	0.15981949
**52**	0.525264767	0.256014323	0.218720909	**152**	0.540531363	0.283363936	0.176104701
**53**	0.491014882	0.286505775	0.222479344	**153**	0.469941387	0.317441976	0.212616637
**54**	0.515034473	0.268014344	0.216951183	**154**	0.487042909	0.312058626	0.200898465
**55**	0.465834412	0.293814705	0.240350883	**155**	0.527026396	0.281223613	0.191749991
**56**	0.477831373	0.299662519	0.222506109	**156**	0.527994044	0.283198703	0.188807253
**57**	0.476329653	0.350386588	0.173283759	**157**	0.513037215	0.253798424	0.233164361
**58**	0.486281845	0.323514806	0.190203349	**158**	0.51237114	0.317956032	0.169672828
**59**	0.507947658	0.258795162	0.23325718	**159**	0.51890398	0.291422702	0.189673319
**60**	0.470394554	0.319725589	0.209879857	**160**	0.492071647	0.304457699	0.203470655
**61**	0.498189894	0.327684083	0.174126023	**161**	0.512470191	0.302136592	0.185393217
**62**	0.475948585	0.328529889	0.195521527	**162**	0.507772459	0.323706044	0.168521497

**63**	0.535905278	0.261280243	0.20281448	**163**	0.502103008	0.267214106	0.230682885
**64**	0.484505332	0.33954331	0.175951359	**164**	0.50300646	0.296658088	0.200335451
**65**	0.511047617	0.283376899	0.205575484	**165**	0.509339972	0.325827261	0.164832766
**66**	0.478861886	0.330746376	0.190391738	**166**	0.519356628	0.312349059	0.168294313
**67**	0.50919298	0.283806283	0.207000737	**167**	0.514519637	0.324772619	0.160707744
**68**	0.551195576	0.275893649	0.172910775	**168**	0.473907907	0.304033569	0.222058524
**69**	0.5266678	0.293099891	0.180232309	**169**	0.506360512	0.302201223	0.191438265
**70**	0.507002671	0.253737511	0.239259818	**170**	0.503988438	0.331247294	0.164764268
**71**	0.460099734	0.323781334	0.216118932	**171**	0.506184539	0.31966932	0.174146141
**72**	0.490306858	0.27873009	0.230963052	**172**	0.480993757	0.310709802	0.208296442
**73**	0.521230816	0.305023189	0.173745995	**173**	0.482891237	0.281669009	0.235439754
**74**	0.551647902	0.292546581	0.155805517	**174**	0.495036072	0.283807667	0.221156262
**75**	0.508422295	0.305371851	0.186205853	**175**	0.491067206	0.304452905	0.204479889
**76**	0.547090005	0.285943584	0.166966411	**176**	0.502184413	0.305668961	0.192146627
**77**	0.472919903	0.310035229	0.217044868	**177**	0.479424058	0.271372277	0.249203665
**78**	0.504634605	0.309124592	0.186240803	**178**	0.494325892	0.325845416	0.179828692
**79**	0.484171252	0.276501853	0.239326895	**179**	0.448558414	0.320310479	0.231131108
**80**	0.499131933	0.333613894	0.167254173	**180**	0.528040289	0.287002352	0.184957359
**81**	0.508309886	0.273655535	0.218034579	**181**	0.477200576	0.287237147	0.235562277
**82**	0.502291356	0.286185297	0.211523347	**182**	0.492525723	0.278589524	0.228884753
**83**	0.496680609	0.307475822	0.195843569	**183**	0.527208429	0.279260532	0.193531039
**84**	0.519976181	0.289415624	0.190608195	**184**	0.493228883	0.256660635	0.250110482
**85**	0.533618846	0.286000721	0.180380433	**185**	0.522377561	0.272446882	0.205175558
**86**	0.482816487	0.290199637	0.226983876	**186**	0.542078817	0.292403199	0.165517984
**87**	0.504266352	0.333977862	0.161755787	**187**	0.480010781	0.288534648	0.231454571
**88**	0.491266889	0.32681342	0.181919691	**188**	0.496149529	0.330341928	0.173508543
**89**	0.489389998	0.271566971	0.239043031	**189**	0.551213091	0.285281638	0.16350527
**90**	0.475973276	0.288263417	0.235763307	**190**	0.523853126	0.283164105	0.19298277
**91**	0.490171853	0.312111004	0.197717143	**191**	0.53858671	0.282047251	0.179366039
**92**	0.510740913	0.303414639	0.185844447	**192**	0.548509528	0.284829882	0.16666059
**93**	0.465410756	0.289931827	0.244657418	**193**	0.486367342	0.292478853	0.221153805
**94**	0.515960759	0.319974717	0.164064524	**194**	0.479659983	0.296595993	0.223744024
**95**	0.475584273	0.320265752	0.204149975	**195**	0.491181098	0.299218711	0.209600191
**96**	0.474251225	0.306288233	0.219460542	**196**	0.46597775	0.295140327	0.238881922
**97**	0.473062039	0.295825263	0.231112698	**197**	0.504210176	0.301645767	0.194144057
**98**	0.466429448	0.315374084	0.218196468	**198**	0.514796848	0.310714846	0.174488306
**99**	0.485197779	0.307052751	0.20774947	**199**	0.484453562	0.276234695	0.239311743
**100**	0.54094722	0.258167081	0.2008857	**200**	0.464547338	0.305874123	0.229578539
				**Base**	**0.5**	**0.3**	**0.2**

Table S11. Resilience metric weight sensitivity analysis (spearman correlation)

Beta	Base_Resilience	Resilience_Mean	Resilience_Std	Resilience_Min	Resilience_Max	Relative_Change_Mean(%)	Relative_Change_Std(%)	Deviation_Mean	Spearman_Corr	Spearman_P
**0.1**	0.86030	0.86013	0.01522	0.82491	0.89046	-0.01923	1.76884	0.03810	-0.05590	0.43171
**0.2**	0.87505	0.87602	0.01364	0.84774	0.90243	0.11070	1.55859	0.03804	0.09790	0.16787
**0.3**	0.88097	0.88050	0.01417	0.84748	0.90969	-0.05329	1.60822	0.04060	-0.02188	0.75848
**0.4**	0.88190	0.88256	0.01425	0.85222	0.91283	0.07489	1.61541	0.04074	0.05371	0.45007
**0.5**	0.88479	0.88375	0.01361	0.85736	0.91125	-0.11748	1.53861	0.03959	0.00900	0.89930
**0.6**	0.88703	0.88711	0.01360	0.85874	0.91651	0.00994	1.53336	0.04048	0.03993	0.57450
**0.7**	0.88838	0.88845	0.01312	0.85855	0.91612	0.00778	1.47726	0.04043	-0.03564	0.61634
**0.8**	0.88951	0.88975	0.01264	0.86344	0.91620	0.02640	1.42108	0.03807	0.03399	0.63274
**0.9**	0.89035	0.88941	0.01313	0.86401	0.91798	-0.10641	1.47446	0.04088	-0.02281	0.74856
**1.0**	0.89013	0.88980	0.01224	0.86626	0.91445	-0.03675	1.37489	0.03795	-0.04217	0.55326
**1.1**	0.89049	0.89218	0.01335	0.86228	0.91568	0.18977	1.49960	0.04055	0.10473	0.13996
**1.2**	0.89175	0.89114	0.01294	0.86511	0.91857	-0.06936	1.45116	0.03894	-0.08803	0.21514
**1.3**	0.89205	0.89215	0.01240	0.86515	0.91581	0.01071	1.38973	0.03911	-0.01073	0.88014
**1.4**	0.89304	0.89443	0.01216	0.86453	0.91788	0.15590	1.36170	0.03767	0.08883	0.21097
**1.5**	0.89342	0.89336	0.01268	0.86645	0.91752	-0.00647	1.41952	0.03783	0.04817	0.49818
**1.6**	0.89313	0.89257	0.01277	0.86596	0.91746	-0.06262	1.43033	0.03961	-0.02687	0.70562
**1.7**	0.89319	0.89291	0.01275	0.86900	0.91895	-0.03093	1.42742	0.04001	-0.02262	0.75050
**1.8**	0.89321	0.89273	0.01272	0.86738	0.91776	-0.05359	1.42429	0.03883	-0.05212	0.46361
**1.9**	0.89388	0.89293	0.01232	0.86850	0.91986	-0.10617	1.37817	0.04059	-0.03039	0.66928
**2.0**	0.89425	0.89345	0.01255	0.86663	0.92162	-0.08993	1.40349	0.04020	-0.08279	0.24383

Table S12. Effect of *β* on cascade failure ratio and network resilience

β	R	OF_avg
0.1	0.8593	0.0243
0.2	0.8740	0.0157
0.3	0.8799	0.0130
0.4	0.8810	0.0116
0.5	0.8834	0.0106
0.6	0.8862	0.0100
0.7	0.8876	0.0096
0.8	0.8888	0.0094
0.9	0.8897	0.0092
1.0	0.8901	0.0092
1.1	0.8899	0.0091
1.2	0.8912	0.0089
1.3	0.8915	0.0088
1.4	0.8925	0.0087
1.5	0.8929	0.0086
1.6	0.8927	0.0085
1.7	0.8927	0.0085
1.8	0.8927	0.0085
1.9	0.8934	0.0084
2.0	0.8938	0.0083

Table S13. Targeted *β* Tuning for Improved Resilience at Critical Nodes

β	No adjustments for all nodes	Critical nodes have 0.1 increase for β	Critical nodes have 0.3 increase for β	Critical nodes have 0.5 increase for β
**0.1**	0.8593	0.8634	0.8766	0.8789
**0.2**	0.8740	0.8754	0.8835	0.8816
**0.3**	0.8799	0.8805	0.8840	0.8822
**0.4**	0.8810	0.8816	0.8840	0.8841
**0.5**	0.8834	0.8837	0.8882	0.8866
**0.6**	0.8862	0.8882	0.8879	0.8880
**0.7**	0.8876	0.8877	0.8890	0.8906
**0.8**	0.8888	0.8889	0.8914	0.8914
**0.9**	0.8897	0.8898	0.8917	0.8917
**1.0**	0.8901	0.8901	0.8900	0.8901
**1.1**	0.8899	0.8900	0.8913	0.8913
**1.2**	0.8912	0.8912	0.8930	0.8916
**1.3**	0.8915	0.8930	0.8926	0.8926
**1.4**	0.8925	0.8925	0.8930	0.8930
**1.5**	0.8929	0.8943	0.8927	0.8927
**1.6**	0.8927	0.8927	0.8928	0.8928
**1.7**	0.8927	0.8927	0.8928	0.8928
**1.8**	0.8927	0.8941	0.8934	0.8934
**1.9**	0.8934	0.8934	0.8938	0.8938
**2.0**	0.8938	0.8938	0.8940	0.8940

S14. Scalability Analysis

S14.1 Runtime Estimation vs. Network Scale

Based on the algorithmic complexity analysis, we estimate the runtime for networks of different scales as follows:

Network Scale (Nodes)	Estimated Runtime	Required Memory	Recommended Configuration
~100	5-10 minutes	< 1 GB	Standard CPU setup
~1,000	30-60 minutes	2-4 GB	Enable approximate computations
~10,000	3-5 hours	8-16 GB	Enable sampling & approximate computations
~100,000	1-2 days	32+ GB	Distributed computing framework

S14.2 Extension to Multi-Layer Networks

The proposed framework can be extended to analyze multi-layer networks through the following approaches:

- Layer-wise Independent Analysis: Each layer can be treated as an independent network for initial intra-layer cascade simulation and feature extraction.
- Inter-layer Coupling Representation: A coupling matrix can be constructed to quantify the dependency strength between nodes across different layers (e.g., based on correlation of eigenvector centrality or flow exchange).
- Unified Representation Learning: Leveraging the inductive learning capability of GraphSAGE to integrate intra-layer features and inter-layer dependencies, generating a cohesive node representation for the entire multi-layer system. This approach maintains the scalability of the original framework while capturing the added complexity of cross-layer interactions.

S14.3 Scalability-Enhanced Algorithm Implementation

(a) Configuration Parameters for Scalability Optimization

```
class ScalabilityConfig:
    """
    Configuration class for adaptive scalability optimization
    based on network size and resource constraints.
    """
    def __init__(self):
        # Network scale thresholds
        self.small_network_threshold = 100        # nodes < 100
        self.medium_network_threshold = 1000    # nodes < 1000
        self.large_network_threshold = 10000    # nodes ≥ 10000

        # Adaptive optimization strategies
        self.enable_betweenness_approximation = False     # Approximate betweenness centrality
        self.enable_cascade_sampling = False                    # Node sampling for cascade simulation
        self.enable_parallel_processing = False               # Parallel computation
        self.max_cascade_nodes = 1000                              # Maximum nodes for cascade simulation
        self.sampling_rate = 0.2                                        # Default sampling ratio

    def auto_configure(self, n_nodes):
        """
```

```
        Automatically configure optimization parameters based on network size.

        Parameters:
        -----------
        n_nodes : int
            Number of nodes in the network

        Returns:
        --------
        self : Updated configuration object
        """
        # Large-scale networks (> 10,000 nodes)
        if n_nodes > self.large_network_threshold:
            self.enable_betweenness_approximation = True
            self.enable_cascade_sampling = True
            self.enable_parallel_processing = True
            self.max_cascade_nodes = min(500, n_nodes // 20)
            self.sampling_rate = 0.1

        # Medium-scale networks (1,000 - 10,000 nodes)
        elif n_nodes > self.medium_network_threshold:
            self.enable_betweenness_approximation = True
            self.enable_cascade_sampling = True
            self.max_cascade_nodes = min(1000, n_nodes // 10)
            self.sampling_rate = 0.2

        # Small networks (< 1,000 nodes)
        else:
            # Use exact computation for small networks
            self.enable_betweenness_approximation = False
            self.enable_cascade_sampling = False
            self.max_cascade_nodes = n_nodes
            self.sampling_rate = 1.0

        return self
```

(b) Optimized Centrality Computation

```
def calculate_scalable_betweenness(G, config=None):
    """
    Scalable betweenness centrality computation with adaptive approximation.

    Parameters:
    -----------
```

```
    G : networkx.Graph
        Input graph
    config : ScalabilityConfig, optional
        Configuration object for scalability optimization

    Returns:
    --------
    dict : Node betweenness centrality values
    """
    # Apply approximation for large networks
    if config and config.enable_betweenness_approximation:
        print("Using approximate betweenness centrality computation...")
        # Adaptive sampling: k = min(100, 10% of nodes)
        k = min(100, max(10, len(G.nodes()) // 10))
        return nx.betweenness_centrality(G, weight='weight', k=k)
    else:
        print("Using exact betweenness centrality computation...")
        return nx.betweenness_centrality(G, weight='weight')
```

(c) Scalable Cascade Failure Simulation

```
def scalable_cascade_simulation(G, beta_values, output_file, config=None):
    """
    Optimized cascade failure simulation with node sampling for large networks.

    Parameters:
    -----------
    G : networkx.Graph
        Input graph
    beta_values : list
        List of β values for simulation
    output_file : str
        Output CSV file path
    config : ScalabilityConfig, optional
        Configuration object for scalability optimization

    Returns:
    --------
    tuple : (DataFrame results, dict node_failure_counts)
    """
    # Node sampling for large networks
    if config and config.enable_cascade_sampling:
        all_nodes = list(G.nodes())
        n_nodes = len(all_nodes)
```

```
        if n_nodes > config.max_cascade_nodes:
            print(f"Network size: {n_nodes} nodes")
            print(f"Applying node sampling: {config.max_cascade_nodes} representative nodes")

            # Select representative nodes based on degree centrality
            degree_centralities = nx.degree_centrality(G)

            # Stratified sampling: proportional representation of high/mid/low-degree nodes
            sorted_nodes = sorted(degree_centralities.items(),
                                  key=lambda x: x[1], reverse=True)

            # Select top-k, middle-k, and random-k nodes
            k = config.max_cascade_nodes // 3
            top_nodes = [node for node, _ in sorted_nodes[:k]]
            middle_nodes = [node for node, _ in sorted_nodes[k:2*k]]
            random_nodes = list(np.random.choice(
                [node for node, _ in sorted_nodes[2*k:]],
                k, replace=False
            ))

            nodes_to_simulate = top_nodes + middle_nodes + random_nodes
            print(f"Selected {len(nodes_to_simulate)} representative nodes "
                  f"(top: {k}, middle: {k}, random: {k})")
        else:
            nodes_to_simulate = all_nodes
    else:
        nodes_to_simulate = list(G.nodes())

    # Call original cascade simulation with sampled nodes
    return cascade_failure_simulation(
        G, beta_values, output_file,
        nodes_to_simulate=nodes_to_simulate
    )
```

(d) Main Function with Adaptive Configuration

```
def scalable_main():
    """
    Main analysis pipeline with adaptive scalability optimization.
    """
    # Initialize paths and parameters
    network_file = 'path/to/network.txt'
    output_dir = 'path/to/output'

    # Read network
```

```
    print("Loading network...")
    G = read_network(network_file)
    n_nodes = len(G.nodes())
    n_edges = len(G.edges())

    # Auto-configure scalability parameters
    config = ScalabilityConfig().auto_configure(n_nodes)

    print(f"\n=== Network Information ===")
    print(f"Nodes: {n_nodes}, Edges: {n_edges}")
    print(f"\n=== Scalability Configuration ===")
    print(f"Betweenness approximation: {config.enable_betweenness_approximation}")
    print(f"Cascade sampling: {config.enable_cascade_sampling}")
    print(f"Parallel processing: {config.enable_parallel_processing}")
    print(f"Max cascade nodes: {config.max_cascade_nodes}")
    print(f"Sampling rate: {config.sampling_rate:.1%}")

    # Optimized centrality computation
    print("\nComputing centrality metrics...")
    betweenness_centralities = calculate_scalable_betweenness(G, config)

    # Optimized cascade simulation
    print("\nRunning cascade failure simulation...")
    beta_values = np.arange(0.1, 2.1, 0.1)
    cascade_results, failure_counts = scalable_cascade_simulation(
        G, beta_values, f'{output_dir}/cascade_results.csv', config
    )

    # GraphSAGE training (already optimized via PyTorch Geometric)
    print("\nTraining GraphSAGE model...")
    # ... [GraphSAGE implementation remains unchanged]

    return {
        'network_stats': {'nodes': n_nodes, 'edges': n_edges},
        'config': config.__dict__,
        'results': cascade_results
    }
```

(e) Performance Metrics and Validation

```
def validate_sampling_accuracy(G, full_results, sampled_results, config):
    """
    Validate the accuracy of sampled cascade simulation.
```

```
Parameters:
-----------
G : networkx.Graph
    Input graph
full_results : DataFrame
    Results from full simulation (ground truth)
sampled_results : DataFrame
    Results from sampled simulation
config : ScalabilityConfig
    Configuration used for sampling

Returns:
--------
dict : Validation metrics
"""
# Calculate key performance indicators
n_nodes = len(G.nodes())

# 1. Node-level accuracy (correlation of failure rates)
full_failure_rates = full_results.groupby('Initial_Node')['Failure_Ratio'].mean()
sampled_failure_rates = sampled_results.groupby('Initial_Node')['Failure_Ratio'].mean()

# Align nodes (sampled subset)
common_nodes = set(full_failure_rates.index).intersection(
    set(sampled_failure_rates.index)
)

if len(common_nodes) > 0:
    full_values = [full_failure_rates[node] for node in common_nodes]
    sampled_values = [sampled_failure_rates[node] for node in common_nodes]

    pearson_corr = np.corrcoef(full_values, sampled_values)[0, 1]
    spearman_corr = spearmanr(full_values, sampled_values)[0]

    # 2. Network-level accuracy (overall failure distribution)
    mae = np.mean(np.abs(np.array(full_values) - np.array(sampled_values)))
    rmse = np.sqrt(np.mean((np.array(full_values) - np.array(sampled_values))**2))

    # 3. Critical node identification accuracy
    # Identify top-k critical nodes from both methods
    k = int(n_nodes * 0.1)  # Top 10%
    full_top_nodes = set(full_failure_rates.nlargest(k).index)
    sampled_top_nodes = set(sampled_failure_rates.nlargest(k).index)
```

```
        overlap = len(full_top_nodes.intersection(sampled_top_nodes)) / k

        validation_metrics = {
            'sampling_rate': config.sampling_rate,
            'nodes_sampled': len(common_nodes),
            'pearson_correlation': pearson_corr,
            'spearman_correlation': spearman_corr,
            'mae': mae,
            'rmse': rmse,
            'top_node_overlap': overlap,
            'computational_speedup': n_nodes / len(common_nodes)
        }

        print(f"\n=== Sampling Validation Results ===")
        print(f"Sampled nodes: {len(common_nodes)}/{n_nodes} "
              f"({len(common_nodes)/n_nodes:.1%})")
        print(f"Pearson correlation: {pearson_corr:.4f}")
        print(f"Spearman correlation: {spearman_corr:.4f}")
        print(f"MAE: {mae:.4f}, RMSE: {rmse:.4f}")
        print(f"Top-node overlap: {overlap:.1%}")
        print(f"Computational speedup: {validation_metrics['computational_speedup']:.1f}x")

        return validation_metrics
```

(f) Configuration Guidelines for Different Network Scales

```
SCALABILITY_GUIDELINES = {
    'small': {
        'description': 'Networks with < 1,000 nodes',
        'config': {
            'enable_betweenness_approximation': False,
            'enable_cascade_sampling': False,
            'enable_parallel_processing': False,
            'max_cascade_nodes': 'all nodes',
            'expected_runtime': '30-60 minutes'
        },
        'recommendation': 'Use exact computation for maximum accuracy'
    },
    'medium': {
        'description': 'Networks with 1,000-10,000 nodes',
        'config': {
            'enable_betweenness_approximation': True,
            'enable_cascade_sampling': True,
            'enable_parallel_processing': 'optional',
```

```
            'max_cascade_nodes': '10-20% of nodes',
            'expected_runtime': '3-5 hours'
        },
        'recommendation': 'Enable sampling for cascade simulation'
    },
    'large': {
        'description': 'Networks with > 10,000 nodes',
        'config': {
            'enable_betweenness_approximation': True,
            'enable_cascade_sampling': True,
            'enable_parallel_processing': True,
            'max_cascade_nodes': '5-10% of nodes',
            'expected_runtime': '1-2 days'
        },
        'recommendation': 'Use all optimization strategies with distributed computing'
    }
}
```

README

Multi-Dimensional Evaluation-Based Graph Neural Network Key Node Identification and Network Resilience Analysis System

I. Project Overview
This project develops a critical node identification system based on a multidimensional evaluation framework. By comparing three mainstream graph neural network models—GCN, GraphSAGE, and GAT—it comprehensively assesses their performance in identifying critical nodes. GraphSAGE is ultimately selected as the optimal model for in-depth resilience analysis. The resilience quantification framework constructs a comprehensive resilience assessment index encompassing three metrics: survival rate, critical node protection, and stability.

II. Seven-Dimensional Evaluation Framework
1. Effect Size
- Metric: Cohen's d
- Purpose: Quantify the standardized difference between critical and ordinary nodes
- Threshold: d<0.2 (negligible), 0.2≤d<0.5 (small), 0.5≤d<0.8 (medium), d≥0.8 (large) (Large)

2. Ranking Consistency
- Metric: Spearman's ρ (with confidence interval)
- Purpose: Evaluate the stability and reliability of node ranking

3. Ranking Quality
- Metric: Spearman's ρ + NDCG@K + F1@K
- Purpose: Balance overall ranking correlation with Top-K prediction accuracy

4. Top-K Identification Accuracy
- Metric: Precision@K, Recall@K, Accuracy@K
- Purpose: Measure precise classification capability for the most important nodes

5. Ranking Agreement
- Metric: Overlap
- Purpose: Compares consistency with alternative ranking methods

6. Computational Efficiency
- Metric: Inference Time
- Purpose: Ensures model practicality for real-world deployment

III. Multi-Model Comparative Analysis
1. Experimental Setup

Parameter	Setting Value
Dataset	USAAir97 Aviation Network (332 nodes, 2126 edges)

Comparison Models	GCN, GraphSAGE, GAT
β Value Range	0.1–2.0, step size 0.1
Evaluation Metrics	*Model Performance Evaluation*: Cohen's d; Spearman's ρ; NDCG@K; F1@K; Precision@K; Overlap; Interference Time. *Critical Node Identification Evaluation*: Analysis of LCC_Ratio and Efficiency changes under deliberate and random attacks. *Node Ranking Stability and Consistency Evaluation*: Spearman correlation coefficient and Jaccard similarity analysis of node rankings between GraphSAGE and baseline methods
Training/Validation/Test Sets	60%/20%/20%

2. Model Selection Criteria

Example: Based on the seven-dimensional evaluation framework, GraphSAGE was selected as the optimal model for the following primary reasons:
- Comprehensive superiority: Achieved top performance across 5 out of 6 evaluation dimensions
- Substantial effect size: Cohen's d reached 1.35, classified as a “large effect size,” indicating the model's ability to clearly distinguish critical nodes from ordinary nodes
- Superior ranking quality: NDCG@33—0.842, excelling in top-K ranking tasks
- Efficient identification capability: F1@33—0.777, achieving optimal balance between precision and recall
- Optimal computational efficiency: Shortest inference time, suitable for real-time deployment

IV. Resilience Composite Metric Construction and Analysis

1. Constructing the resilience composite evaluation metric R, encompassing survival rate SR, critical node protection CNP (TOP10%), and cascade stability CS
- Sensitivity analysis of resilience metric weights
2. Resilience Analysis
- Analysis of cascade failure proportion S variation with redundancy coefficient β
- Analysis of resilience R variation with redundancy coefficient β
- Analysis of the relationship between targeted critical node β adjustment strategies (β+0.1/0.3/0.5) and network resilience R

V. Project Structure

```
Graph Neural Network Key Node Identification System/
├── 1_Model Comparison Analysis/
│ ├── gcn_model.py # GCN model implementation
│ ├── graphsage_model.py # GraphSAGE model implementation
│ ├── gat_model.py # GAT model implementation
│ └── model_comparison.py # Multi-model comparative analysis
├── 2_Optimal Model Deployment/
│ ├── PCA_beta_ablation.py # GraphSAGE model PCA and β ablation experiments
│ ├── cascade_simulation.py # Cascade failure simulation
│ └── resilience_analysis.py # Network resilience analysis
├── 3_Visualization and Reporting/
│ ├── comparison_visualization.py # Model comparison visualization
│ ├── performance_report.py # Performance report generation
│ └── resilience_visualization.py # Resilience analysis visualization
```

```
├── data/
│ ├── inf_USAAir97_332Data.txt # Network data
│ └── results/ # Multi-level result output
│ ├── model_comparison/ # Model comparison results
│ ├── graphsage_optimal/ # GraphSAGE optimal model results
│ └── resilience_analysis/ # Resilience analysis results
├── Main program entry point/
│ ├── step1_model_comparison.py # Step 1: Model Comparison
│ ├── step2_optimal_model.py # Step 2: Optimal Model Analysis
│ └── step3_resilience_analysis.py # Step 3: Resilience Evaluation
└── Public Libraries/ # New: Public library directory
  ├── __init__.py # Package initialization file
  ├── gnn_libs.py # Graph Neural Network libraries (PyTorch, etc.)
  ├── network_reading_processing.py # Network reading and processing functions
  ├── public_utilities.py # General utility functions (statistical tools, etc.)
  └── cascade_topology_libs.py # Cascade failure and topology metric libraries
```

#### VI. Software Architecture

Core Modules

1. Data Preprocessing Module
- Network file reading and parsing
- Node centrality calculation (degree, closeness, betweenness, eigenvector)
- Information entropy calculation

2. Cascading Failure Simulation Module
- Capacity-load-based cascading failure model
- Multi-β parameter scanning
- Failure statistics and metric calculation

3. GNN Model Module
- Graph Convolutional Neural Network architecture
- Pairwise Ranking Loss function
- Stratified sampling for training/validation/test partitioning

4. Evaluation and Analysis Module
- Effect Size Analysis (Cohen's d)
- Bootstrap Confidence Interval Calculation
- Multiple Evaluation Metrics (Spearman Correlation Coefficient, Precision@K, NDCG@K, etc.)

5. Visualization Module
- Result Chart Generation
- Effect Size Visualization
- Attack Strategy Comparison

Technology Stack
- Deep Learning Framework: PyTorch 1.13+, PyTorch Geometric 2.2+
- Network Analysis: NetworkX, scipy
- Data Processing: pandas, numpy
- Machine Learning: scikit-learn
- Visualization: matplotlib, seaborn
- Statistical Analysis: Bootstrap (Bootstrap), Effect Size Analysis

VII. Installation Guide
1. Environment Requirements
- Python 3.8+
- PyTorch 1.13.0+
- CUDA 11.6+ (if using GPU)

2. Installation Steps

- **Method 1: Using conda (Recommended)**

(1) Clone the repository
git clone https://gitee.com/your-username/critical-node-identification.git
cd critical-node-identification

(2) Create a conda environment
conda create -n cascading-failure python=3.9
conda activate cascading-failure

(3) Install PyTorch (based on your CUDA version)
conda install pytorch torchvision torchaudio pytorch-cuda=11.7 -c pytorch -c nvidia

(4) Install PyTorch Geometric
conda install pyg -c pyg

(5) Install other dependencies
pip install -r requirements.txt

- **Method 2: Using pip**

(1) Clone the repository
git clone https://gitee.com/your-username/critical-node-identification.git
cd critical-node-identification

(2) Create a virtual environment
python -m venv venv
source venv/bin/activate # Linux/Mac

or venv\Scripts\activate # Windows

(3) Install dependencies
pip install torch torchvision torchaudio --index-url https://download.pytorch.org/whl/cu117
pip install torch-scatter torch-sparse torch-cluster torch-spline-conv torch-geometric -f https://data.pyg.org/whl/torch-1.13.0+cu117.html
pip install -r requirements.txt

3. Verify Installation
- python -c “import torch; import torch_geometric; print(‘PyTorch version:’, torch.__version__); print(‘PyG version:’, torch_geometric.__version__)”

VIII. Quick Start
1. Prepare Data

Place network files in the specified directory:
- Default data path
data/raw/inf_USAAir97_332Data.txt

- Data format
- Per line: Source node   Target node   [Weight]
1   2   0.5
1   3   0.8
2   3   1.0

2. Configuration File

Modify config/settings.py:

```
NETWORK_FILE = “data/raw/inf_USAAir97_332Data.txt”
OUTPUT_DIR = “results/”
BETA_VALUES = [0.1, 0.2, 0.3, 0.4, 0.5, 0.6, 0.7, 0.8, 0.9, 1.0]
GCN_PARAMS = {
“hidden_channels”: 64,
“num_layers”: 2,
“dropout”: 0.5,
“learning_rate”: 0.005,
“epochs”: 200,
‘loss_type’: “PairwiseRanking”
}
```

3. Parameter Description

- Command-line arguments:

```
python main.py --network_file “data/raw/your_network.txt” \
--output_dir “your_results/” \
--beta_start 0.1 \
--beta_end 2.0 \
--beta_step 0.1 \
--gcn_hidden 128 \
--gcn_layers 3 \
--gcn_epochs 300 \
--top_percent 0.15
```

IX. Usage Workflow

Step 1: Multi-Model Comparative Analysis

- Run model comparison experiment

```
python step1_model_comparison.py --dataset USAAIr97 --models GCN GraphSAGE GAT
```

- Output results:

1. Table of evaluation metrics across seven dimensions for each model
2. Model comparison radar chart
3. Comprehensive scoring report

Step 2: In-depth Analysis of Optimal Model

- Conduct in-depth analysis based on the selected GraphSAGE model

```
python step2_optimal_model.py --model GraphSAGE --beta_range 0.1 2.0 0.1
```

- Output results:

1. GraphSAGE model performance (including PCA and β ablation studies)
2. Predictive capability analysis
3. Critical node identification results

Step 3: Network Resilience Assessment

- Conduct resilience assessment based on identified critical nodes

```
python step3_resilience_analysis.py --critical_nodes top_10_percent --beta_adjustments 0.1 0.3 0.5
```

- Output results:

1. Comprehensive resilience index R and sensitivity analysis of resilience index weights
2. Relationship between resilience index R and β, and analysis of cascade failure proportion S versus β
3. Key node β sensitivity analysis

X. Visualization Output

The system automatically generates the following key charts:

- Seven-dimensional evaluation comparison table (CSV) - Compares the performance of three models across each dimension

- PCA and β ablation comparison table (CSV) - Compares the performance of the optimal model GraphSAGE across seven dimensions before and after ablation experiments
- Model comparison bar chart - Displays model comparisons across each metric
- Comprehensive Score Heatmap - Performance of models at different β values
- Resilience Metric Trend Chart - Trends of resilience metric R and cascading failure proportion S with β value
- Resilience Metric Weight Sensitivity Analysis Chart - Variation in resilience value R under fluctuations of three resilience metric weights
- Critical Node Sensitivity Analysis - Resilience changes under different β adjustments

XI. Detailed Usage Instructions (Performance Comparison of GCN/GraphSAGE/GAT Models, Using GCN as Example)

1. Network File Formats

Supported network file formats:
- Edge list: node1 node2 [weight]
- Adjacency Matrix: CSV format
- GML/GraphML: NetworkX-supported formats

2. Cascade Failure Simulation

```
from src.cascade_simulation import cascade_failure_simulation

# Run cascade failure simulation
results = cascade_failure_simulation(
G, # NetworkX graph object
beta_values=(0.1, 2.1, 0.1), # β values
output_file="results/cascade_results.csv"
)
```

3. GCN Model Training

```
from src.gcn_model import train_gcn_model, GCNModel

# Train GCN model
model, metrics = train_gcn_model(
data, # PyG Data object
hidden_channels=64,
epochs=200,
loss_type="PairwiseRanking", # MSE, Huber, SmoothL1, PairwiseRanking
```

```
margin=1.0, # Pairwise Ranking Loss margin parameter
patience=15 # early stopping patience
)
```

4. Effect Size Analysis

```
from src.effect_size_analysis import bootstrap_effect_size_analysis

# Perform effect size analysis
results = bootstrap_effect_size_analysis(
group1_scores, # List of key node scores
group2_scores, # List of non-critical node scores
group1_name="Critical Node",
group2_name="Non-Critical Node",
n_bootstrap=1000, # Number of bootstrap resamples
confidence_level=0.95
)
```

5. Attack Strategy Evaluation

```
from src.attack_simulation import (
targeted_attack_simulation,
random_attack_simulation,
compare_attack_strategies
)

# Run attack simulations
gcn_attack_results, robustness_gcn = targeted_attack_simulation(G, gcn_scores)
random_results, robustness_random = random_attack_simulation(G)

# Compare attack strategies
comparison_results = compare_attack_strategies(
attack_results={
"GCN Attack": gcn_attack_results,
"Random Attack": random_results
},
output_dir="results/attack_comparison/"
)
```

6. Custom Configuration

Create config/custom_config.yaml:

```
network:

    file_path: "data/raw/my_network.txt"
    is_weighted: true
    is_directed: false

cascade:

    beta_values: [0.05, 0.1, 0.15, 0.2, 0.25]
    capacity_model: "betweenness" # betweenness, degree, eigenvector
    redistribution_method: "eigenvector" # eigenvector, degree, equal

gcn:

    features: ["degree", "closeness", "betweenness", "entropy", "clustering"]
    hidden_layers: [64, 32]
    activation: "relu"
    optimizer: 'adam'
    learning_rate_schedule: "reduce_on_plateau"

evaluation:

    metrics: ["spearman", "precision@k", "recall@k", 'ndcg', "jaccard"]
    top_k_percentages: [0.05, 0.1, 0.15, 0.2]
    bootstrap_iterations: 5000
    confidence_level: 0.99
```

Run with custom configuration:

```
python main.py --config config/custom_config.yaml
```

7. Output Results

- Generated file structure

```
results/
├── data/ # Data files
│   ├── cascade_results.csv # Cascade failure results
│   ├── node_metrics.csv # Node metrics
│   ├── detailed_failure_data.csv # Detailed failure data
```

```
│ └── gcn_results_effectsize.csv # GCN scoring results
├── figures/ # Visualization charts
│ ├── effect_size_visualization.png # Effect size visualization
│ ├── gcn_vs_true_scatter_effectsize.png # GCN vs true values scatter plot
│ ├── correlation_heatmap_effectsize.png # Correlation Heatmap
│ ├── multiple_attack_comparison_lcc.png # Multiple Attack Strategy Comparison
│ └── loss_and_lr_curve.png # Training Loss Curve
├── models/ # Model Files
│ └── best_gcn_model.pth # Best GCN Model
└── evaluation/ # Evaluation Results
├── gcn_evaluation_results_effectsize.csv
├── jaccard_similarity_matrix.csv
└── time_statistics.csv
```

- **Key Metric Explanations**

(1) Effect Size Metric

Here is the list text Cohen's d: Measures the magnitude of difference between two groups

- $|d| < 0.2$: Negligible effect;
- $0.2 \leq |d| < 0.5$: Small effect;
- $0.5 \leq |d| < 0.8$: Moderate effect;
- $|d| \geq 0.8$: Large effect.

(2) Confidence Interval: 95% confidence interval calculated using bootstrapping

e.g., [-0.15, 0.35] containing 0 indicates no significant difference

(3) Ranking Quality Metrics

- Spearman correlation coefficient: Consistency between GCN ranking and true ranking
- Precision@K: Proportion of correctly identified nodes among the top K
- NDCG@K: Discounted cumulative gain considering ranking position
- Jaccard similarity: Overlap between identification results of different methods

(4) Network robustness metrics

- Robustness coefficient: Area under the LCC curve; higher values indicate greater robustness
- Failure rate: Proportion of nodes ultimately failing in the network
- Cascade steps: Number of time steps the cascade process persists

- **Example of Result Interpretation**

Summary of Running Results:

- Total runtime: 356.42 seconds
- GCN Model Training Time: 42.17 seconds
- GCN Model Inference Time: 0.023 seconds

Effect Size Analysis Summary:

- Effect size distinguishing critical from non-critical nodes via GCN scores: 1.2436
- Effect size interpretation: Large effect size
- Significant difference in GCN scores between critical and non-critical nodes

Evaluation Metrics:

- Spearman correlation: 0.8724 (95% confidence interval: [0.8451, 0.8932])
- Is correlation significant: Yes
- Precision@33: 0.8485
- NDCG@33: 0.9217
- Overlap rate: 0.7576

XII. Advanced Features

1. Batch Experiments

- Run parameter sweeps

```
python scripts/parameter_sweep.py \
--network_files data/raw/*.txt \
--beta_ranges “0.1:0.5:0.1” “0.1:1.0:0.2” \
--gcn_hidden 32 64 128 \
--gcn_layers 2 3 4 \
--output_dir results/parameter_sweep/
```

2. Cross-Validation

- Run 5-fold cross-validation

```
python scripts/cross_validation.py \
--folds 5 \
--stratified true \
--repeats 10 \
--output_dir results/cross_validation/
```

3. Sensitivity Analysis

- Analyze the impact of different parameters on results

```
python scripts/sensitivity_analysis.py \
--parameter beta \
--range 0.1 2.0 0.1 \
--metrics failure_ratio robustness_coefficient \
--output_dir results/sensitivity/
```

4. Network Scale Expansion

- Process large-scale networks

```
python main.py \
--network_file data/raw/large_network.txt \
--optimization_level high \
--batch_size 1000 \
--use_sparse true \
--num_workers 4
```

XIII. Testing and Validation

Test Directory Structure

```
Graph Neural Network Key Node Identification System/
├── tests/
│ ├── test_reproducibility.py # Reproducibility verification
│ ├── test_model_consistency.py # Model consistency verification
│ └── benchmark.py # Performance benchmarking
├── utils/
│ └── data_preparation.py # Test data preparation
├── models/
│ └── graphsage_model.py # GraphSAGE model
└── results/ # Results output directory
```

1. Run Test Suite

- Test GraphSAGE model module
python graphsage_model.py

- Test resilience analysis module
python resilience_analysis.py

- Test performance reporting module
python performance_report.py

- Test the resilience visualization module
python resilience_visualization.py

2. Reproducibility Verification

- Verify result reproducibility (using the same random seed)
python tests/test_reproducibility.py --seed 42 --output-dir results/reproducibility

3. Model Consistency Verification

```
python tests/test_model_consistency.py \
--model1 results/models/model_v1.pth \
--model2 results/models/model_v2.pth \
--tolerance 1e-6 \
--output-dir results/model_consistency
```

4. Performance Benchmarking

```
python tests/benchmark.py \
--network_sizes 100 500 1000 5000 \
--repeat 5 \
--output_file results/benchmark/benchmark.json \
--seed 42
```

XIV. Academic Citation
If using this system, please cite:

```
@article{Citation2025 (replace after paper publication),
title={Critical Node Identification and Resilience Analysis against Cascading Failures},
author={Anqi Liu},
journal={PLOS One},
year={2025},
doi={10.xxxx/xxxx} (replace after paper publication)
}
```

** Paper Template: **

If you use this code for research and publish a paper, we recommend including the following description in the Methods section:

"We employed a graph neural network-based critical node identification framework (Gitee link), which integrates multiple network centrality metrics, utilizes Pairwise Ranking Loss for model training, and performs statistical testing via Cohen's d effect size and bootstrap confidence intervals. "

Features Under Development
- Support for directed graphs and dynamic networks
- Integration of additional GNN architectures (GAT, GraphSAGE)
- Addition of real-time visualization interface
- Support for distributed computing
- Provision of REST API interface
- Addition of more network types (social networks, biological networks, etc.)

Support & Contact

- Gitee Issues: Submit questions
- Email: 992890775@qq.com
- Discussion Forum: Project Gitee Discussion Forum
- Project Homepage: https://gitee.com/anqi_Liu666/critical-node-GNN-comparison
- Documentation Updated: December 2025

Academic Collaboration

If you wish to use this framework in your research or explore collaboration, please contact us via email. We are happy to:
- Provide technical support and consultation
- Assist with custom development
- Discuss potential collaborative research projects

License
This project is licensed under the MIT License - see the LICENSE file for details.

Terms of Use
- Commercial use, modification, and distribution permitted
- Original license statement must be retained
- Authors assume no liability for consequences of using this code
- Attribution required when citing

Citation Requirements
If you use this code in your research, please:
- Cite this repository in your paper's Data/Methods section
- Acknowledge this project's contributions in your acknowledgments section
- Consider co-authorship (if contributions are significant)

Acknowledgments

We thank the following open-source projects for their contributions:
- PyTorch Geometric - Graph Neural Network library
- NetworkX - Complex network analysis
- scikit-learn - Machine learning tools

Data Sources
- US airline network data (1997)
- Other publicly available complex network datasets

Research Support

We acknowledge the work of researchers in related fields, with special thanks to:
- The complex network research community
- The graph neural network research community
- The statistical methods research community

If this project helps your research, please show your support with a ⭐ Star!

https://gitee.com/AnqiLiu/paper-reproduction-2025/badge/star.svg?theme=dark
https://gitee.com/AnqiLiu/paper-reproduction-2025/badge/fork.svg?theme=dark

Last updated: December 2025
