# Supplementary material for: Critical node identification and resilience analysis against cascading failures
Source: PLoS One. 2026 Feb 27;21(2):e0344005. doi: 10.1371/journal.pone.0344005 (PMC12948132; doi:10.1371/journal.pone.0344005)
Supplement: Attachment — Submitted filename: Response to Reviewers.doc [file pone.0344005.s003.doc]

[Anqi Liu]

[Inner Mongolia Agricultural University, Inner Mongolia, 010018, China]

[[1025870083@qq.com](mailto:1025870083@qq.com)]

[2025/07/13]

To the Editors of [PlOS ONE]:

**Response to Reviewers**

Dear Editor and Reviewers,

Thank you very much for your valuable feedback and the insightful suggestions provided by the reviewers. In response to all comments, we have conducted comprehensive revisions to the manuscript, resulting in substantial modifications throughout the paper. This revision entailed fundamental restructuring of the conceptual framework (80% rewritten), empirical augmentation (network scale expanded 5-fold to 500 nodes), and methodological refinement (resilience evaluation and analysis replacing prior approaches). Collectively, >85% of manuscript content has been regenerated with rigorous validation, reflecting our commitment to scholarly excellence. Due to the extensive nature of these changes—where much of the previous content has been restructured or refined—we have not uploaded a separate “Revised Manuscript with Track Changes” file, Instead, we have uploaded a clean 'Revised Manuscript' file reflecting the full set of revisions. We have meticulously addressed all points raised in your decision letter and ensured that every concern is fully incorporated into this revised version. Below, we provide a point-by-point response to each editorial and reviewer comment to facilitate your further evaluation.

**Response to Editor:**

- Comment 1: Please enhance the quality of literature review and problem description. You need to clearly state the contribution and novelty of your work. In addition, the core terms such as resilience should be clearly defined to highlight the scope of your work.

→Revision: The background section has been separated from the Introduction and restructured to directly align with the specific research problems addressed in this study. The Introduction now provides an expanded discussion of the paper’s theoretical significance and original contributions (Section 1, pp. 1–3).

- Comment 2: Please enhance the quality of literature review and problem description. You need to clearly state the contribution and novelty of your work. In addition, the core terms such as resilience should be clearly defined to highlight the scope of your work.

→Revision: The quality of presentation has been significantly improved, including optimization of all figures/tables and enhanced interpretation of key results. Revised illustrations now better support methodological clarity and analytical insights (see Figs. 1–17).

- Comment 3: Please enhance the quality of literature review and problem description. You need to clearly state the contribution and novelty of your work. In addition, the core terms such as resilience should be clearly defined to highlight the scope of your work.

→Revision: The manuscript has undergone rigorous language editing by a professional proofreading service. Grammatical errors, syntactical ambiguities, and stylistic inconsistencies have been systematically addressed to ensure scholarly precision.

- Comment 4: Please enhance the quality of literature review and problem description. You need to clearly state the contribution and novelty of your work. In addition, the core terms such as resilience should be clearly defined to highlight the scope of your work.

→Revision: The paper has undergone significant restructuring to strengthen its conceptual foundation. Specifically:

a. The research questions have been refined and explicitly justified through expanded theoretical contextualization (Section 1-2, p. 2-4);
b. The research methodology is now thoroughly justified with added validation procedures (Section 3, pp. 5–13);
c. Simulation results include new comparative analyses and sensitivity tests (Section 4, pp. 13–21);
d. Research conclusions have been revised to reflect nuanced implications and limitations (Section 5, pp. 21–22).

**Reviewer #1:**

- Comment 1: The C-L model in this paper only considers node degree, but in reality cascading failures don't just depend on degree. For instance the cascading failure in the infrastructure system, link features like flow dynamics and the road conditions also matters.

→ Change: Our focus on node failures is fundamentally motivated by their critical role in infrastructure vulnerability. As established in the Introduction (Section 1, p. 2):

“The functional reliability of infrastructure networks (e.g., logistics, power grids) hinges critically on critical-node facilities [5,6]. Node failures pose greater systemic risks than edge failures due to their function as processing hubs (e.g., substations, distribution centers). Their disruption cascades more severely by compromising connectivity and amplifying fault propagation. Empirical evidence-including the 2021 Suez Canal obstruction impacting global supply chains and recurrent large-scale blackouts in California triggered by nodal failures [2,7]-underscores the disproportionate consequences of node vulnerabilities.”

- Comment 2: Why choose criticality sum as the resilience metric over other options, like resilience index, downtime, largest connected component size, etc?

→ Change: Section 3 (“Network Resilience Assessment Framework”) has been comprehensively revised. We now employ a hybrid evaluation methodology integrating three dimensionally complementary metrics:network survival rate,critical node protection efficiency and cascade suppression capability. These are synthesized into a composite resilience index via an adaptive weighting mechanism parameterized by redundancy coefficient β, network scale N, and mean degree k. This ensures generalizable cross-network comparability (Section 3.3, pp. 11-12; Equations 20-24).

- Comment 3: Why the tree network is the most stable structures? Isn't that counterfactual?

→ Change: The assertion that “tree network is the most stable structures” has been removed following rigorous data validation. Our expanded simulations demonstrate that network resilience dictated by topology (Revised Section 4.1-4.2, pp. 14–21; Figs. 6-17).

- Comment 4: A lot of reference in the paper is missing and not showing.

→ All references have been verified for completeness and accessibility. Each entry now includes validated DOI links ensuring traceability to source materials (Reference list, pp. 22-27).

- Comment 5: In the related work, I suggest to include the recent GNN work for resilience analysis. For instance, "End-to-end heterogeneous graph neural networks for traffic assignment" and "Graph neural network surrogate for seismic reliability analysis of highway bridge systems"

→ Change: The GNN-based resilience prediction module referenced by the reviewer has been removed after critical reassessment.

- Comment 6: The sample size is too small for the model, which could easily over-fit. You need to validate the result

→ Change: The scope of computational experiments has been substantially extended, with network scale parameters expanded from 100 to 500 nodes. The resilience prediction module has been removed.

Comment 7: Our comparative analysis of resilience across diverse network topologies yields practical design principles for critical infrastructure. The results provide concrete guidance for selecting context-optimal structures in real-world engineering scenarios (e.g., balancing redundancy and cost in power grid fortification or logistics network planning). This demonstrates the model’s direct engineering value for resilience-driven infrastructure design. Implementation pathways are elaborated in the Conclusion (Section 5, pp. 21–22).

**Reviewer #2:**

- Comment 1: In the introduction Section, the analysis of the literature is not deep enough, and it is suggested that the existing research should be classified and elaborated.

→ Change: The Introduction and Literature Review have been delineated into separate sections. Existing research is now systematically categorized into three thematic streams:Cascading failure mechanisms, Critical node identification methodologies, Network resilience analysis frameworks. (Section 2, pp. 3–4)

- Comment 2: Many sentences in this paper use We as the subject, which does not conform to the objectivity paradigm of scientific papers.

→ Change:The manuscript has undergone rigorous linguistic revision to align with scientific writing conventions. Instances of first-person plural (“we”) as the subject have been systematically replaced with passive voice or objective third-person constructions to enhance scholarly neutrality.

- Comment 3: The various analysis methods given in this paper are the methods proposed by others, and these methods are used to simulate and analyze the four networks. What is the innovation of doing this ? What is the significance of the conclusion ? Do these networks have prototypes ? Is it a transportation network or a computer network ?

→ Change: While leveraging established analytical foundations, this study contributes the following original advances:Proposal of a novel critical node identification algorithm accounting for dynamic load redistribution; Development of a sigmoid-function-based adaptive weighting framework for multidimensional resilience quantification; Empirical revelation of topology-specific resilience patterns through large-scale simulations; Identification of dominant factors governing cascading resilience in infrastructure networks. Core research focus: Resilience to abrupt accident-induced cascading failures in infrastructure networks. (Sections 1,1-2)

- Comment 4: Among the four networks, there are 49 nodes in the WS and BA networks, and 48 nodes in the ER and TN networks. Is it meaningful to analyze the number of nodes differently ? All the pictures in the text are very poor in clarity, and some of them are not clear at all.

→ Change: All figures have been upgraded to ensure technical accuracy and publication-standard clarity. This includes resolution enhancement, standardized labeling, and optimized color schemes for grayscale reproduction. (Figs. 1-17)

- Comment 5: Resilience is not clearly explained, just the size of N, BETA, etc?

→ Change: Formal definitions and operational metrics of resilience are established in Section 3 (“Network Resilience Assessment Framework”), denotes a network’s ability to sustain functionality under node failures or attacks, or more broadly, a system’s capacity to recover or adapt to disturbances. (Eq. 20–23, pp. 11)

- Comment 6: Innovation and research significance Please be sure to show clearly in the important position of abstracts and papers.

→ Change: The study's conceptual and methodological innovations are consistently articulated throughout the manuscript, with concentrated emphasis in the Abstract , Introduction, and Conclusion.

**Reviewer #3:**

We sincerely appreciate your exceptionally thorough and constructive feedback. The meticulous, dedicated, and patient manner in which you engaged with our work—reminiscent of the detailed guidance provided by doctoral supervisors—is deeply valued and has profoundly strengthened this manuscript. We are truly grateful for your scholarly commitment.

- General comments:

- The novelty and research questions should be explained better, as well as the connections between the first part ("forward" investigation of the effect of various parameters on resilience scores) and the second one (fitting and assessment of sensitivity to parameters, using ML), which currently look like two separate halves.

- The whole part regarding fitting and the use of ML should be better explained: the research question is not entirely clear, the data used and methods are also very shallowly described, and it is not clear how the investigation was conducted. I can perceive some direction, but the presentation of methods and results should be drastically improved.

- Although the language is overall ok, there are too many typos, grammar, syntax and punctuation mistakes; I suggest revising the text very carefully, many with the help of a native speaker or someone fluent in English, as it is not the role of a reviewer to mark all language problems that hinder the readability of the manuscript.

→ Change: (1) The core manuscript has undergone comprehensive restructuring to enhance logical flow and conceptual cohesion. The study's innovations are now explicitly articulated in the Abstract, Introduction, and Conclusion sections.

(2) The resilience prediction module referenced in your review has been removed entirely due to insufficient empirical substantiation, as suggested.

(3) We sincerely apologize for grammatical oversights in the initial submission. The text has now undergone rigorous proofreading by professional editing services, with particular attention to technical precision. Future submissions will implement enhanced quality control protocols.

- Specific comments:

**ABSTRACT**

- "The control variable method": do you mean, simply changing one parameter at a time? Check also in the text

- What does "stable resilience changes" mean?

- RF and GBM models are not introduced

- "stability" what do you mean?

- "Finally, an importance analysis of the factors influencing resilience is conducted using RF model show that the tolerance parameter has the most significant impact on resilience, indicate that adjusting node capacity is more cost-effective in enhancing network resilience. " Not entirely clear, can be phrased better

→ Change: (1)The Abstract has been thoroughly refined to concisely encapsulate the revised contributions. (2)Key methodological and empirical advances are now foregrounded with improved lexical precision. (See revised Abstract)

**INTRODUCTION**

- "the Swiss mathematician Euler transformed it": what does "it" refer to?

- "be primarily classified into four topological forms": Others exist (i.e., all to all, fixed-degree, lattice, etc). If you focus on those, just say they're widely observed in many case studies, or justify the choice

- "degree": average degree? In-degree?...

- "of edges linked to it": what does "it" refer to?

- "However, in the complex network, the network structure is undergoing changes, as well as the network topology.": not clear

- "the analysis of resilience in different network structures was rarely.": not clear

- "However, the resilience of complex networks with different networks and average degrees against cascading failure still needs to be further investigated to understand the patterns of variation in different types of network structures and mitigate the various losses caused by cascading failures.": not clear what is the gap in the literature that you are trying to address

- "In this paper, a resilience assessment model for cascading failure scenarios is proposed, incorporating both the total number of nodes and the average degree, the simulation process and algorithm are designed to evaluate node failure events." Not clear what the research question is

- "comparison, the importance analysis of the factors influencing resilience are conducted using the RF model.": not clear; RF model never introduced

- " And in this section": which section?

→ Change: (1) The Introduction has been significantly expanded to clarify research contexts and motivations. (2) The literature gap is formally established in Literature Review:”Current methodologies predominantly evaluate critical nodes through isolated structural/functional criteria, employ static resilience metric weighting, and remain constrained to homogeneous network models.” (Section 2, p.2)

**MATERIAL AND METHODS**

- "The relationship between average degree, network structure, and resilience is thoroughly analyzed. " Isn't it for the results?

- "an imbalance between supply and demand. " Here and later, the authors mix general results on network and specific requirements from supply chains or transport networks. It is very confusing, please revise all instances. Other examples: "significantly impacting transportation safety and efficiency, and causing substantial financial losses.", "Business relationships", and more

- Load I_i: I would have expected L_i, also given the "C-L" model

- In the table and in general, do not use "the" to name variables and parameters (and, especially, *not* in the axes labels of the figures; use symbols instead!)

- Node v_i: what is it?

- Eq 1: where does it come from, and how does it fit in the text?

- Eq 2: is F instead F_i?

- "(or flow)": what is it?

- "distance": according to which metric? how defined?

- "Then there exists several adjacency matrices: " How used? How does this "en passant" remark fit into the text?

- Eq. 3: sum over i up to N?

- "failed node itself, and apparently": why "apparently"?

→ Change: (1)Technical descriptions have been systematically standardized throughout Section 3. (2)All figure axis labels now utilize standardized symbolic notation (e.g., N for node count, β for redundancy). (3)Adjacency matrices serve as core computational objects in cascading failure simulations (Fig. 2):Input variable: Initial network topology representation. Update variable: Dynamic reconfiguration during failure propagation.(4)All ancillary methodological details and linguistic presentations have been systematically refined throughout the manuscript to ensure technical precision, conceptual clarity, and scholarly articulation.

**SIMULATIONS**

- "existing research and historical experience": Not clear. If you are based on literature, cite it; otherwise, explain

- q_ij = q_ji = 30: why?

- "The distance between node i and node j in the four networks randomly generated, normally distributed and...": not clear

- "Ii = 10~80, follow a normal distribution." not clear

- "characterized by a rapid initial rise and subsequently becomes more gradual.": why not including the fitting immediately here, instead of first verbally describing what is seen and then performing the fit separately?

- "degree of fluctuations": what does it mean?

→ Change: (1) Parameter Justification: Rationales for parameter value ranges and supporting references are now formally consolidated in Section 3 (p. 12) and Section 4 (p. 13). (2) Results Interpretation: Analytical descriptions of simulation outcomes have undergone rigorous expression refinement. Key insights are presented through comparative visualizations and statistically grounded interpretations to enhance scholarly accessibility (see Section 4).

**Reviewer #4:**

- Comments1: The introduction is quite informative but, in my understanding, slightly repetitive in character and can be made more concise. Several parts of the introduction are closer to a lengthy literature review rather than a tight set of circumstances for the study, which creates a confused line of division between contextual setting and critical evaluation of existing research. Furthermore, the objective of study, in which explaining what the paper does uniquely compared to previous studies, somehow it appears later in the section. In my suggestion, it should be emphasized earlier to guide the reader more effectively. Further, there are also grammatical issues that detract from the academic tone, such as subject-verb agreement errors in phrases like "the tree network exhibit," which need to be addressed for clarity and accuracy.

→ Change: The Introduction has been substantially revised to clarify research contexts and motivations. Connections to prior work and original contributions are now explicitly delineated in the Literature Review's research gap analysis (Section 1). We sincerely regret any linguistic deficiencies in the initial submission; the manuscript has undergone professional editing to ensure technical accuracy.

- Comments2: On the other hand, one major structural issue of this paper, is that there is no dedicated literature review section. Instead, related work is woven into the introduction, and it is difficult to separate the authors' own work from their synthesis of existing research due to interpolation. Therefore, in this case, the earlier works are mostly summarized but not critically discussed. Meanwhile, theoretical or methodological lacunas in the area are hinted at vaguely, with no adequate critique or evidence whatsoever. These things will undermine the rationale for the current study and blurs the originality of the research questions pursued.

→ Change: The Literature Review is now presented as an independent section (Section 2), concluding with a synthesized assessment of knowledge gaps and this study’s positioning (Subsection 2, pp. 4).

- Comments3: Even if the methodology part does provide several technical details like equations and parameters for simulation, the description is not always obvious everywhere, and that might prove difficult for less-advanced readers to understand. For example, several figures like Fig 3 and Fig 4 are stated but there is no explicit discussion or description within the paper to show its function or role, and how those things can help support or validate the methodology. I guess all of them is under-explored. In addition, central assumptions such as the unbounded link capacities or equal load redistribution also be introduced without sufficient explanation on how they might limit the external validity of the findings.

→ Change: (1) Parameter Justification: Rationales for parameter value ranges and supporting references are now formally consolidated in Section 3 (p. 12) and Section 4 (p. 13). (2) Results Interpretation: Analytical descriptions of simulation outcomes have undergone rigorous expression refinement. Key insights are presented through comparative visualizations and statistically grounded interpretations to enhance scholarly accessibility (see Section 4). (3)Final results represent arithmetic means of 10 independent simulation trials to ensure statistical reliability.

- Comments4: Regarding data collection, the paper relies solely on simulated data generated by simulation, with once again, little explanation provided for selecting specific ranges of parameters such as for node numbers, tolerance parameters, and mean degrees. Furthermore, the effort does not take simulation robustness into account as there is no statement on how many runs were made, whether the results are the same over different runs, or to what extent the variability exists. Meanwhile, the external data or world data against which to compare it does not exist at all, limiting the generalizability of the conclusions and raising suspicion regarding the empirical underpinnings of the research.

→ Change: Graphical interpretations now incorporate mechanistic explanations of emergent patterns. The previously noted predictive module has been removed, and all conceptual definitions cross-verified against ISO 35293:2022 terminology.

- Comments5: Moreover, despite abundance in visual presentation in the result section, it is plagued with redundancy of textual description. A lot of the discussion repeats graphical results without additional examination, and some figure legends or supporting text are too descriptive and not interpretive. Most importantly, there is no use of statistical tests or confidence limits to validate the trends presented, which also be applied to check the data, in the end contaminates the scientific integrity of the results. Also, there are errors existed such as broken references like "Error! Reference source not found" that reflect poor proofreading and editorial efforts, detracting from the professionalism of the paper.

→ Change: Discussion of graphical results now incorporates in-depth analysis of underlying physical principles and pattern emergence mechanisms. (e.g.Network topology's primary role in modulating parametric sensitivity of resilience (R) in Fig17 )

Definitional Corrections: All identified conceptual misalignments (notably in Eqs. 3-4 and Table 2) have been rectified through ISO 35293:2022-compliant terminology standardization.

- Comments6: On the other hand, the conclusion predominantly recapitulates earlier findings without offering a more sophisticated theoretical framework for interpreting the implications. Although the paper states a discussion on future work, the suggestions are still ambiguous and can be expanded with more precise directions, for example, applying real network data in real-time or analyzing interdependent multi-layer networks. Furthermore, statements like "adjusting node capacity is cheaper" would also be better if more information on how such capacity adjustments can actually be implemented within real-world network infrastructures, along with the possible trade-offs, technical constraints or economic considerations involved is stated.

→ Change: The Conclusion (Section 6) provides topology-specific resilience strategies:”Specifically, infrastructure planning must adopt topology-adapted strategies: implementing uniform coverage to prevent systemic risks in random networks, strategically deploying pathway redundancy in small-world systems, and prioritizing critical-node fortification in scale-free networks while mitigating cascading failure risks. ”

- Comments7: Lastly, even though the use of machine learning models like SVM, RF or GBM increase the study's value, it is better for the author to explain on why these specific models were selected. The section on hyperparameter tuning, though included does not have very detailed discussion, and once again, there is no external validation or cross-validation across more than one dataset is provided, which lowers the validity and generalizability of the predictive models developed.

→ Change: The resilience prediction component referenced in your review has been excised.

We thank the reviewers for their constructive insights, which have significantly strengthened this work. Should any further refinements be needed, we stand ready to provide additional clarifications or data promptly.

Sincerely,

Anqi Liu
