# Supplementary material for: Critical node identification and resilience analysis against cascading failures
Source: PLoS One. 2026 Feb 27;21(2):e0344005. doi: 10.1371/journal.pone.0344005 (PMC12948132; doi:10.1371/journal.pone.0344005)
Supplement: Attachment — Submitted filename: Rebuttal Letter.doc [file pone.0344005.s005.doc]

**Rebuttal Letter**

**Manuscript Title:** [Critical Node Identification and Resilience Analysis against Cascading Failures]

**Manuscript ID:** [PONE-D-25-14454R2]

**Authors: [**Anqi Liu**]**

**Journal: [**PLoS ONE**]**

**Date: [**2025/12/10**]**

Dear Editor and Reviewers,

We express our sincere gratitude to the editors and reviewers for dedicating their time, offering valuable feedback, and providing constructive comments on our manuscript titled "[**Critical Node Identification and Resilience Analysis against Cascading Failures**]" (Manuscript ID: **[PONE-D-25-14454R3]**). We have meticulously considered all the comments and implemented substantial revisions to enhance the quality of the manuscript.

In response to the reviewers' comments, we have thoroughly revised the manuscript, incorporating substantial improvements across theoretical, empirical, and methodological dimensions. Approximately 70% of the content has been regenerated and rigorously validated, reflecting our commitment to enhancing the scholarly quality of the paper.

The key revisions are summarized as follows:

- **Theoretical Integration:** The conceptual framework has been restructured (≈30% rewritten) to systematically integrate the Artime et al.(2024)' [16] study throughout the introduction, methodology, and discussion, strengthening both the theoretical foundation and practical relevance of the work.
- **Methodological Refinement:** The model construction has been redesigned, with evaluation metrics now including Cohen's d and bootstrap testing (*n_bootstrap=1000, confidence_level=0.95, random_seed=42*) to ensure statistical robustness. Additionally, we conducted ablation studies on PCA and *β* parameters, along with a sensitivity analysis of resilience assessment weights. These experiments offer deeper insights into the functional importance of key model components, ensuring that the identified critical nodes are both accurate and methodologically interpretable.
- **Scalability and Large-Scale Network Applications:** A dedicated section has been added to discuss the scalability of the proposed framework, demonstrating its feasibility and efficiency when applied to large-scale networks. This enhances the practical utility and generalizability of our research.
- **Extended Discussion:** The Discussions section has been substantially expanded to provide a more nuanced interpretation of results, clearly articulate the scholarly and practical contributions, acknowledge limitations, and outline specific directions for future research. This strengthens the depth and reflexivity of the manuscript.
- **Visual Presentation and Empirical Support**: The figures have been systematically revised and expanded to align with and strengthen the textual improvements. Key updates include a refined Fig.1 in the methodology section and the addition of new Fig. 6, 7, 9(a), and 10. These new figures visually present the critical results from the added ablation studies, model performance evaluations, and sensitivity analyses, offering direct and clear graphical support for the manuscript's enhanced methodological narrative and findings.

These comprehensive revisions have significantly improved the clarity, validity, and contribution of the manuscript. We believe the revised version addresses all concerns raised by the reviewers and substantially advances the scholarly discussion in this field.

In the following, we present a point-by-point response to the reviewers' comments, accompanied by explanations of the modifications made in the revised manuscript. In response to all the comments, we have carried out comprehensive revisions of the manuscript, leading to substantial alterations throughout the paper.

We have carefully addressed all the points raised in your decision letter and ensured that every concern is fully integrated into this revised version. Below, we provide a point-by-point response to each editorial and reviewer comment to facilitate your further assessment.

**# Response to Editor:**

- **Comment :** Please review your reference list to ensure that it is complete and correct. If you have cited papers that have been retracted, please include the rationale for doing so in the manuscript text, or remove these references and replace them with relevant current references. Any changes to the reference list should be mentioned in the rebuttal letter that accompanies your revised manuscript. If you need to cite a retracted article, indicate the article’s retracted status in the References list and also include a citation and full reference for the retraction notice.

**→Response:**

Thank you for the reminder and your thorough review. We have conducted a comprehensive verification and systematic update of the references cited in the manuscript to ensure their completeness, accuracy, and timeliness. The specific revisions are as follows:

- **Reference Replacement and Update:** References **10, 17, 21, 22, 34, 39,** and **43** from the original manuscript have been replaced with more recent and authoritative literature in the relevant field. This strengthens the scholarly foundation and supporting evidence for the corresponding arguments.
- **Addition of Key Methodological Literature:** New references **44, 45,** and **46** have been added. These sources focus on "node entropy" as a core metric for identifying critical nodes in complex networks, providing more direct and solid literature support for the methodological framework of this study.
- **Addition of Literature on Parameter Justification:** New references **54, 55,** and **56** have been included. These publications systematically discuss methods and rationales for determining multi-indicator weights in resilience assessment, offering clear theoretical and empirical references for the weight parameter settings in our research.

All cited references have been re-checked for their publication status, confirming that **no retracted articles have been cited**. The updated reference list demonstrates significant improvement in terms of completeness, relevance, and authority. All corresponding changes have been fully incorporated into the revised manuscript.

Thank you once again for your valuable feedback.

**Reviewer #3:**

We sincerely thank you for your thorough and constructive feedback, as well as for the kind acknowledgment of the substantial effort invested in revising the manuscript. We are grateful for the your time and insightful comments, which have further helped us refine the paper. Below, we provide a point-by-point response to the raised issues and confirm that all corresponding corrections have been made in the revised manuscript.

- **Comment 1**: Sec. "Mechanisms of cascading failure propagation", "As Fig.1 shows, when node i (i=1,2, …, n)"... hare and later: do you actually refer to Fig. 2?

→ **Response & Action Taken**: You are correct. The text "As Fig.1 shows…" in the section "Mechanisms of cascading failure propagation" should refer to Fig. 2. This has been corrected in the manuscript (**Page 7, Line 26**).

- **Comment 2**: Below Eq. 15: My PDF viewer cannot read the sybol after \beta: what is that?

→ **Response & Action Taken**: The symbol following *β* is the "*β ϵ B* " (**Page 13, Line 3**), and entirely text —"Where *β* ϵ *B* is the redundancy coefficient of the network, *OF_β_* = *N_failed_* / *N_total_* represents the overall failure proportion of the system under redundancy coefficient *β*. *N_failed_* denotes the total number of failed nodes, and *N_total_* is the total number of nodes in the network. *T* is the set of top-K critical nodes identified by the GNN model. *M* is the total number of simulations, *f_i_* is the number of failures of node *V_i_* over M simulations. *σ_β_* is the standard deviation of cascade steps under *β*. *B* is the set of all considered redundancy coefficient *β*. The terms *w_sr_, w_cnp_, w_cs_* represent the weights for the three indicators.". To avoid any display issues, we have ensured the font is embedded correctly in the revised version for clarity.

- **Comment 3**: p_value of what?

→ **Response & Action Taken**: In response to the request for clarification on the reported p_value, we have enhanced our statistical analysis by replacing it with Cohen's d (to quantify effect size) and bootstrap testing (*n_bootstrap=1000, confidence_level=0.95, random_seed=42*) to ensure robust inference. This update, detailed in the revised **Simulations and Results sections**, provides a more comprehensive and reliable assessment of performance differences between the compared methods. (**Page 16, Line 14, Table 3**).

- **Comment 4**: In general, I would suggest to mild down the tone of the findings and of the use of GraphSAGE: althugh it slightly improved with comparison to other methods such as GCN, the difference is not tremendous, both in the identification of critical nodes and in computing time (it is x4, not orders of magnitude). It sufficees to present the findings without sliping into boastful language.

→ **Response & Action Taken**: We appreciate this valuable suggestion and agree that the language should remain objective. We have moderated the tone throughout the **Results and Discussion sections**. Descriptions such as "dramatically outperforms” or "significantly superior” have been replaced with more measured phrasing. The discussion now focuses on presenting the observed trends without overstatement.

- **Comment 5**: Section "efffects of *β*.": many variables are writeen with a strange "_" in the pedix. Revise?

→ **Response & Action Taken**: Thank you for catching this typesetting issue. The strange underscores in variable subscripts (e.g., w_sr, w_cs...) were artifacts from manuscript conversion. They have been revised to proper subscript formatting (e.g., *w_sr_*, *w_cnp_*, and *w_cs_* ) throughout the section.(**Page 20, Line 10-38**).

- **Comment 6**: Not entirely clear how the found fixed allocation ratio of 2:1 between w_sr and w_cs translates into "universal resilience patterns". It is an interesting finding, but Gao et al mostly considered bifurcation-driven failures, which I don't see how they connect with the case here. Can you clarify? Is this remark necessary?

→ **Response & Action Taken**: We thank the reviewer for raising this important point, which highlights a needed clarification in our terminology and the conceptual linkage. We appreciate the opportunity to refine our argument. The reviewer is correct that the term "universal resilience patterns" can be misinterpreted and that the failure mechanisms in our work (cascading, load-based) differ technically from the bifurcation-driven failures analyzed by Gao et al. Our intention in citing their work was not to equate the mechanisms, but to reference their broader conceptual framework which proposes that quantifiable, recurrent relationships (or "patterns") can characterize resilience structure across different systems. Inspired by this general premise, we investigated whether a stable, empirical relationship between key resilience component weights—specifically the ratio between survivability (*w_sr_*) and cascading stability ( *w_cs_* )—would emerge from our analysis.

To substantiate the robustness and relevance of the observed approximate 2:1 ratio within our specific context, we have conducted a comprehensive sensitivity analysis (detailed in the Section **Effects of β and Targeted Tuning on Resilience** (**Page 20-21**)). The analysis confirms that:

- The final resilience metric *R* is largely insensitive to moderate perturbations around the proposed weights, with relative changes consistently below 1.5%.
- The ranking of network states based on *R* remains stable under these perturbations.

This demonstrates that the weight set, and by extension the observed ratio, provides a stable and reliable basis for comparative resilience assessment within the defined framework and case studies. In the revised manuscript, we have therefore:

- Refined our terminology, replacing the phrase "universal resilience patterns" with more precise language such as "a stable and empirically-observed weight relationship" or "a consistent parametric pattern within our modeling framework."
- Clarified the conceptual connection to Gao et al. by explicitly stating that while the failure mechanisms differ, their work motivates the broader search for quantifiable structural relationships in resilience analysis. We then focus the discussion on the empirical evidence for this relationship derived from our own sensitivity analysis.
- Strengthened the justification by integrating the outcomes of our sensitivity analysis, which directly supports the practical stability and validity of the chosen weights and the observed ratio.

**Reviewer #7:**

We sincerely thank you for your rigorous evaluation and for providing these detailed, actionable suggestions. We appreciate the recognition of improvements in the revised version and are pleased to address the remaining fundamental concerns. The points raised have been instrumental in driving the final and most substantial enhancements to our manuscript's rigor, clarity, and scholarly foundation. Below is our point-by-point response and a summary of the actions taken.

- **Comment 1:** Correct the cascading failure model: Add normalization factor to Eq. 2: ΔF_i->j = (load to distribute) × (w_ij / Σ_k w_ik). Current formulation violates load conservation..

→ **Response & Action Taken**: We gratefully acknowledge the reviewer for identifying this critical technical oversight. The reviewer is absolutely correct that the original formulation did not ensure load conservation during redistribution. We have corrected Equation (2) precisely as suggested, incorporating the normalization factor (w_ij /Σ_k w_ik). This revision guarantees the physical consistency and mathematical accuracy of our cascading failure simulation model. The updated description is now present in the **Mechanisms of cascading failure propagation** section (**Page 8, Line 13**).

- **Comment 2:** Strengthen statistical rigor: Replace implausible p-values with appropriate effect sizes (Cohen's d) and bootstrapped significance, Add ablation studies for each component (PCA, entropy, \beta), Conduct sensitivity analysis on resilience metric weights..

→ **Response & Action Taken**: We agree entirely that a more nuanced and robust statistical presentation is required. In direct response:

**(1) Updated Statistical Reporting**: We have replaced generic p-values with Cohen's d for effect size quantification and implemented bootstrap significance testing (with n_bootstrap=1000, confidence_level=0.95, and a fixed random_seed=42). This is now standard in the **Simulations and Results** section (**Page 15-17, Fig. 6, S1; Table 3, S4** ).

**(2) Comprehensive Ablation Studies**: We thank the reviewer for raising the important point regarding ablation studies for all model components. Ablation studies on model components has been added. It systematically evaluates the contribution of the PCA module and the *β* parameter to the final model performance, confirming the functional importance of each (**Page 17-18**). These studies confirm the distinct and significant contribution of each to the model's final performance and interpretability.

Regarding the node entropy metric, we made a deliberate methodological design choice not to treat it as an optional, "ablated" component in the same manner. Our rationale, which we have now clarified in the revised manuscript, is as follows:

- Established Theoretical Foundation: The node information entropy is not a novel, untested parameter of our model but a well-established foundational metric in complex network analysis for assessing node importance. As elaborated in the "Theoretical Background" section and supported by the newly added references [44-46], entropy-based measures are rigorously derived from information theory and have been widely validated across numerous studies (e.g., Wu et al., 2023; Huang et al., 2024; Maji, 2025) for their efficacy in identifying critical nodes by capturing the diversity and uncertainty of information flow within a node's neighborhood.(**Page 9, Line 11-20**)
- Role as a Core Input Feature, Not a Tunable Hyperparameter: In our framework, node entropy is calculated as a primary input feature that characterizes the intrinsic structural and functional property of each node. It serves as a crucial, non-redundant information source that complements simpler metrics like degree centrality. The ablation studies for PCA and *β* directly test our framework's design choices on how to process and synthesize these input features (including entropy). Ablating the entropy metric itself would not test a model design decision but rather remove a fundamental, theory-grounded descriptor of the system, effectively changing the problem formulation.
- Focus on Integration and Novelty: The core methodological contribution of our work lies in the novel integration and processing of established metrics (like entropy) within a dynamic, GNN-based framework that also accounts for load and cascade dynamics. The ablation studies on PCA and *β* are therefore targeted precisely at validating the necessity of our novel integration steps, which is the key advance beyond the state-of-the-art cited in references [44-46].

**(3) Detailed Sensitivity Analysis**: Sensitivity analysis of resilience weights has been introduced. It empirically tests the stability of our composite resilience metric *R* against perturbations in the weight parameters (*w_sr_*, *w_cnp_*, and *w_cs_* ), demonstrating the robustness of our evaluation framework and the empirical validity of the chosen weight set.(**Page 20, Line 10-38, Fig. 10**)

We believe this approach ensures rigorous validation of our novel contributions while building responsibly upon established theoretical pillars. We thank the reviewer for prompting this clarification, which has improved the precision of our methodological exposition.

- **Comment 3:** Scalability: Reviewer #5 mentioned multilayer/hierarchical networks – the authors added USAir97 but did not address computational scalability for large networks or complex topologies. Address this point.

→ **Response & Action Taken**: We thank the reviewer for the valuable comment on scalability. In response, we have added a dedicated subsection "**Scalability and Large-Scale Network Applications**" (**Page 23-24**) to the manuscript. This addition provides a rigorous, three-part analysis: (1) Theoretical scalability is established by analyzing the near-linear time complexity of the core algorithms (GraphSAGE inference and parallel cascade simulation); (2) Empirical validation is demonstrated through efficiency metrics on the USAir97 network and performance projections for networks with up to 10,000 nodes; and (3) Pathways for complex topologies are explicitly outlined, including strategies to extend the framework to multilayer networks via hypergraph mapping or tensor representations **(Supplementary Material: S14)**. This structured discussion substantiates the framework's applicability to large-scale systems while charting clear directions for future extension beyond the current study's scope. The revision can be found in the indicated section of the updated manuscript.

- **Comment 4:** Reproducibility. Provide full reproducibility package:Public repository with all code, hyperparameters, and preprocessing scripts, deposit raw simulation outputs in a public repository, Include random seeds, software versions (PyTorch).

→ **Response & Action Taken**: Thank you for raising this important point. We strongly support the principles of open and reproducible research and have taken the following comprehensive measures to fully address your request:

- **Complete Reproducibility Package**: We have created a detailed code repository containing all source code, comprehensive configuration files (specifying all hyperparameters and random seeds), preprocessing scripts for the USAir97 dataset, and complete software environment specifications (including versions of key libraries such as PyTorch).
- **Access Instructions**: As the manuscript is currently under revision, to prevent premature disclosure of the work, the repository is currently set to **private**. To ensure you can review the materials, we can grant you access in the following way:

*	Please provide a valid email address, and we will add it as a **collaborator with read-only access** to the repository, allowing you to view all files.

*	We have included a detailed project documentation (README) in the **Supplementary Materials** of the manuscript, which outlines the repository structure, dependencies, and execution steps.

- **Commitment to Public Release**: We sincerely commit that this repository will be made **fully public** immediately upon final acceptance of the paper, to facilitate verification by the academic community and support future research.
- **Repository Link:** https://gitee.com/anqi_Liu666/paper-reproduction-2025
- **Comment 5:** Taxonomy: the manuscript does not position itself within the resilience taxonomy framework Artime proposes, missing opportunity for theoretical grounding. Address this point.

→ **Response & Action Taken**: We sincerely thank the reviewer for highlighting the need to better position our work within an established theoretical framework. In direct response, we have fundamentally revised the manuscript to systematically integrate the resilience taxonomy proposed by Artime et al. [16]. This integration is not superficial but forms the core conceptual architecture of our study.

As detailed in the revised **Introduction** (**Page 2-3**), we explicitly adopt the taxonomic perspective of Artime et al., which deconstructs network resilience into three interrelated dimensions: 1) Structural Robustness, 2) Strategic Protection Efficacy, and 3) Dynamic Stability. Our primary contribution is to operationalize this theoretical tripartite framework into a concrete, hybrid assessment methodology.

Accordingly, in the **Methodology** (**Page 13, line 10-26**) section, the three core metrics of our proposed framework—Survival Rate (*SR*), Critical Node Protection Rate (*CNP*), and Cascade Stability (*CS*)—are designed to directly quantify these three theoretical dimensions, respectively. Specifically, *SR* anchors the structural robustness dimension via percolation theory; *CNP*, calculated using GraphSAGE-identified key nodes, aligns with the strategic protection (network disintegration) dimension; and *CS* measures the dynamic stability dimension through cascade simulation variance. This creates a coherent "metric-theory" mapping, transforming the conceptual taxonomy into an actionable, nested assessment system.

Furthermore, in the **Discussions** (**Page 24, line 35-40**) , we interpret our empirical findings—such as the superior performance of GraphSAGE for key node identification—through this theoretical lens. We reference Artime et al. to support that GraphSAGE's inductive mechanism is particularly suited for the strategic protection dimension, thereby linking our methodological choice back to the established taxonomy. This integration ensures that our work is not merely applied but is theoretically grounded, providing a multidimensional and process-oriented framework for resilience analysis that directly addresses the limitation of unidimensional approaches noted in the literature.

We are confident that these comprehensive revisions have decisively addressed all the reviewer's concerns, substantially elevating the manuscript's methodological soundness, reproducibility, theoretical depth, and clarity of contribution. We are deeply grateful for the reviewer's expert guidance, which has been invaluable in shaping this final, much-improved version.

Sincerely,

[Anqi Liu]

[Inner Mongolia Agricultural University, Inner Mongolia, 010018, China]

[[1025870083@qq.com](mailto:1025870083@qq.com)]
