# Supplementary material for: Critical node identification and resilience analysis against cascading failures
Source: PLoS One. 2026 Feb 27;21(2):e0344005. doi: 10.1371/journal.pone.0344005 (PMC12948132; doi:10.1371/journal.pone.0344005)
Supplement: Attachment — Submitted filename: Response_to_Reviewers_auresp_4.doc [file pone.0344005.s006.doc]

**Response to Editor**

**Manuscript Title:** [Critical Node Identification and Resilience Analysis against Cascading Failures]

**Manuscript ID:** [PONE-D-25-14454R4]

**Authors: [**Anqi Liu**]**

**Journal: [**PLOS ONE**]**

**Date: [**1/25/2026**]**

Dear Editor,

Thank you for your guidance regarding the reference list and final revisions for our manuscript titled "**Critical Node Identification and Resilience Analysis against Cascading Failures**]" (Manuscript ID: **[PONE-D-25-14454R3]**). We have carefully addressed each suggestion to enhance clarity, accessibility, and rigor. Below is a summary of revisions:

**1. Reference list verification:** We have meticulously reviewed all citations against both *Web of Science* and *Elsevier databases* to ensure completeness, accuracy, and compliance with journal formatting standards. All references are currently active and uncorrected. All references were generated using *EndNote software* to guarantee consistent formatting and eliminate manual errors. The reference list reflects this standardized output.

**2. Retracted articles:** Our literature search confirms that no retracted articles were cited in this revision. Should any future citations involve retracted works, we will explicitly note their status per your guidelines.

**3. Final revisions:** In accordance with the reviewers' final minor suggestions, we have implemented all requested edits (detailed in the point-by-point response). The manuscript now fully addresses all feedback. The key revisions are summarized as follows:

- **Abstract refinement for multidisciplinary readership:** The abstract was revised to reduce technical density by adding contextual background and clarifying result significance. Explanatory text and data detailing the specific performance parameters of the GraphSAGE model and the advantages of PCA dimensionality reduction have been incorporated. This ensures accessibility to non-specialist readers while retaining technical precision.
- **Clarification of Table S1 (Pages 14-15)** : Table S1, previously embedded as unannotated text, now includes a concise introductory sentence explaining its purpose: corresponding data for centrality metrics of all nodes, enabling readers to quickly assess robustness without consulting supplementary details.
- **Full Naming of Metrics (First Introduction):** Metrics NDCG@K and F1@K were updated to display their full names upon first mention in abstract and Section ''**Evaluation Metrics**''(**Page1, Line 24-25; Page16, Line 486-487**, as ''Normalized Discounted Cumulative Gain at TOP-K (NDCG@K)'' and ''F1-score at TOP-K (F1@K),'' ensuring clarity for readers unfamiliar with acronyms.
- **Explicit Link Between Figures and Supplementary Tables:** The phrase ''as illustrated in Fig. 9 (Tables S8 and S9)'' was revised to clarify relationships: ''Tables S8 and S9: corresponding to the list data for Fig. 9(a) and 9(b), which present critical node rankings in list format, comparing GraphSAGE with baseline methods, thereby enabling granular validation of the conclusions derived from Fig.9.'' (**Page20, Line 574-576**)

These revisions strengthen the manuscript's readability and transparency. Detailed point-by-point responses and tracked changes are provided in the accompanying **Response to Reviewers**. We believe the revised version better aligns with the journal's scope and reviewer expectations.

Thank you again for your guidance.

Sincerely,

Anqi Liu

Corresponding Author, School of Energy and Transportation Engineering, Inner Mongolia Agricultural University

**Response to Reviewers**

**Reviewer #3:**

Thank you sincerely for your constructive feedback and positive assessment of our revisions. We greatly appreciate your recognition of the strengthened contributions of this work. Your suggestions have helped us further improve the clarity and accessibility of the manuscript. Below we provide a point-by-point response detailing the modifications made in accordance with your recommendations.

**Comment 1**: The abstract is very technical, and the results are there presented with little context and significance I would suggest to be clear and accessible to multidisciplinary readerships. Also, "GraphSAGE outperforms peers": do you mean it outperforms alternative DL models? and "PCS enhances discriminative power" of what?

→ **Response & Action Taken**: We agree that the abstract required greater accessibility for interdisciplinary readers. We have revised it to:

- **Add contextual significance:** Explicitly state the real-world implications of our findings.
- **Clarify ambiguous terms:** 1) Replaced "GraphSAGE outperforms peers"with "Systematic evaluation indicates that the GraphSAGE model delivers the best overall performance in critical node identification. Its results exhibit high consistency with supervised signals (Spearman's correlation coefficient of 0.822), achieving a Normalized Discounted Cumulative Gain at Top-K (NDCG@K) of 0.918, an F1 Score at Top-K (F1@K) of 0.879, and a Top-K accuracy of 0.879. Its inference efficiency (0.002 s) is comparable to GCN and significantly outperforms GAT, meeting the demands of real-time analysis for large-scale networks".

2) Revised "PCA enhances discriminative power"to "After feature dimension reduction via principal component analysis (PCA), the model's discriminative power further improved, with effect size (Cohen's d) increasing by approximately 4% without efficiency loss, validating the effectiveness of scientific dimension reduction".

- **Highlight broader impact:** Emphasized how our framework bridges graph representation learning and practical applications. "This strategy provides an efficient path for system fortification under resource constraints. The research framework proposed in this paper provides interpretable and scalable theoretical and methodological support for vulnerability assessment and resilience enhancement in critical infrastructure. The validated GraphSAGE model and ''targeted reinforcement'' strategy are particularly suitable for risk prevention and resource optimization in major infrastructure systems requiring dynamic analysis and rapid response, such as transportation and power grids."

**Revised Abstract (Page 1)**

→ Ensuring the robustness and resilience of critical infrastructure networks such as transportation and energy systems is a core security challenge for modern societies. Vulnerabilities in these networks often concentrate on a small number of critical nodes, whose failure can trigger catastrophic cascading failures. Therefore, accurately identifying critical nodes and formulating effective reinforcement strategies are crucial for enhancing the overall defense capability of the system. Existing graph neural network (GNN)-based methods often rely on topological centrality metrics, neglecting the distribution of node information and the impacts of cascading failures. To bridge this gap, this study constructs a comprehensive analytical framework (TEC-GNN, Topology-Entropy-Cascading Graph Neural Network) integrating graph neural networks, feature engineering, and resilience assessment. It aims to address two core questions: which graph neural network model is most suitable for critical node identification, and how to enhance network resilience by regulating redundant resource allocation. Systematic evaluation indicates that the GraphSAGE model delivers the best overall performance in critical node identification. Its results exhibit high consistency with supervised signals (Spearman's correlation coefficient of 0.822), achieving a Normalized Discounted Cumulative Gain at Top-K (NDCG@K) of 0.918, an F1 Score at Top-K (F1@K) of 0.879, and a Top-K accuracy of 0.879. Its inference efficiency (0.002 s) is comparable to GCN and significantly outperforms GAT, meeting the demands of real-time analysis for large-scale networks. After feature dimension reduction via principal component analysis (PCA), the model's discriminative power further improved, with effect size (Cohen's d) increasing by approximately 4% without efficiency loss, validating the effectiveness of scientific dimension reduction. The model's accuracy was robustly validated through attack experiments: selectively removing the top 10% critical nodes identified by GraphSAGE reduced the network's largest connected component ratio (LCC_Ratio) to approximately 0.4, severely impairing network functionality. When the removal rate reached 20% (equivalent to 60% removal in random attacks), the network became nearly paralyzed. Another core finding reveals the complex nonlinear regulatory mechanism of redundancy coefficient β on network resilience. The resilience metric R exhibits clear diminishing marginal returns with increasing β: R rises rapidly as β increases from 0 to 0.5, then slows significantly with fluctuations thereafter. Based on this, the study proposes a ''precision reinforcement'' strategy: enhancing redundancy allocation only for critical nodes identified by GraphSAGE enables low-cost resilience improvement (e.g., R increases from 0.874 to 0.883). This strategy provides an efficient path for system fortification under resource constraints. The research framework proposed in this paper provides interpretable and scalable theoretical and methodological support for vulnerability assessment and resilience enhancement in critical infrastructure. The validated GraphSAGE model and ''targeted reinforcement'' strategy are particularly suitable for risk prevention and resource optimization in major infrastructure systems requiring dynamic analysis and rapid response, such as transportation and power grids.

**Comment 2**: Page 14 and 15: Table S1 is included as text, but it is not clear which information it may contain. Please give a quick reference as of why a reader should look it up.

→ **Response & Action Taken**: We have added explicit references to Table S1 in the main text:

Inserted a sentence ''Table S1: Corresponding data for centrality metrics of all nodes''. (**Page 15, Line 445**).

- **Comment 3**: NDCG@K and F1@K: I suggest displaying their naming in fulll, and not just the acronym, the first time they are introduced.

→ **Response & Action Taken**: Metrics NDCG@K and F1@K were updated to display their full names upon first mention in abstract and Section ''Evaluation Metrics''(**Page1, Line 24-25; Page16, Line 486-487**, as ''Normalized Discounted Cumulative Gain at TOP-K (NDCG@K)'' and ''F1-score at TOP-K (F1@K),'' ensuring clarity for readers unfamiliar with acronyms.

- **Comment 4**: "As illustrated in Fig. 9 (Tables S8 and S9)" please better specify how the tables can be useful and their relationship with the figures

→ **Response & Action Taken**: We have clarified the connection between Figure 9 and Supplementary Tables S8-S9:Added annotations ''Tables S8 and S9: corresponding to the list data for Fig. 9(a) and 9(b), which present critical node rankings in list format, comparing GraphSAGE with baseline methods, thereby enabling granular validation of the conclusions derived from Fig.9.'' (**Page 20, Line 574-576**).

We deeply value your insightful suggestions, which have substantially enhanced the readability and rigor of our manuscript. All revisions have been highlighted in the revised manuscript and summarized in the response letter. Thank you again for your time and expertise.

Sincerely,

Anqi Liu

Inner Mongolia Agricultural University, Inner Mongolia, 010018, China

[1025870083@qq.com](mailto:1025870083@qq.com)
